# HiViPore: a highly viable in-flow compression for a one-step cell mechanoporation in microfluidics to induce a free delivery of nano- macro-cargoes

**DOI:** 10.1186/s12951-024-02730-y

**Published:** 2024-07-27

**Authors:** Maria Isabella Maremonti, Valeria Panzetta, Paolo Antonio Netti, Filippo Causa

**Affiliations:** 1 Interdisciplinary Research Centre on Biomaterials (CRIB), Dipartimento di Ingegneria Chimica, dei Materiali e della Produzione Industriale, University of Naples “Federico II”, Naples, 80125 Italy; 2 Center for Advanced Biomaterials for Healthcare@CRIB, Istituto Italiano di Tecnologia, Naples, 80125 Italy

**Keywords:** Intracellular delivery, Microfluidics, Contactless compression, Mechanoporation, Membrane tension, Nano-cargoes, Macro-cargoes

## Abstract

**Background:**

Among mechanoporation techniques for intracellular delivery, microfluidic approaches succeed in high delivery efficiency and throughput. However, especially the entry of large cargoes (e.g. DNA origami, mRNAs, organic/inorganic nanoparticles) is currently impaired since it requires large cell membrane pores with the need to apply multi-step processes and high forces, dramatically reducing cell viability.

**Results:**

Here, HiViPore presents as a microfluidic viscoelastic contactless compression for one-step cell mechanoporation to produce large pores while preserving high cell viability. Inducing an increase of curvature at the equatorial region of cells, formation of a pore with a size of ~ 1 μm is obtained. The poration is coupled to an increase of membrane tension, measured as a raised fluorescence lifetime of 12% of a planarizable push-pull fluorescent probe (Flipper-TR) labelling the cell plasma membrane. Importantly, the local disruptions of cell membrane are transient and non-invasive, with a complete recovery of cell integrity and functions in ~ 10 min. As result, HiViPore guarantees cell viability as high as ~ 90%. In such conditions, an endocytic-free diffusion of large nanoparticles is obtained with typical size up to 500 nm and with a delivery efficiency up to 12 times higher than not-treated cells.

**Conclusions:**

The proposed one-step contactless mechanoporation results in an efficient and safe approach for advancing intracellular delivery strategies. In detail, HiViPore solves the issues of low cell viability when multiple steps of poration are required to obtain large pores across the cell plasma membrane. Moreover, the compression uses a versatile, low-cost, biocompatible viscoelastic fluid, thus also optimizing the operational costs. With HiViPore, we aim to propose an easy-to-use microfluidic device to a wide range of users, involved in biomedical research, imaging techniques and nanotechnology for intracellular delivery applications in cell engineering.

**Supplementary Information:**

The online version contains supplementary material available at 10.1186/s12951-024-02730-y.

## Background

Intracellular delivery of exogenous material is a critical procedure for molecular imaging techniques, drug discovery and cell engineering [1]. Cargoes of interest are highly variable in size, shape, architecture, and chemical properties. Indeed, the size scale ranges over more than three orders of magnitude, from small (~ 1 nm) to large nanomaterials (~ 1 μm). That range encompasses a diversity of origins, from antibodies, quantum dots, RNPs, RNAs (like siRNA or miRNA) to molecules like DNAs origami, mRNAs or synthetic materials such as carbon nanotubes, and organic/inorganic nanoparticles (NPs) [2, 3]. For delivery and intracellular applications, current approaches involve either the packaging of the cargo with specific carriers -which undergo uptake into endocytic routes- or the poration of the cell plasma membrane (PM) to facilitate the entry of the nanomaterial [3, 4]. By this means, the delivery occurs through the created discontinuities, escaping the endocytic routes [2]. Indeed, carrier-mediated endocytosis approaches often hinder the biological and functional activity of the introduced nanomaterial because of multiple transport barriers such as vesicle trapping, cytoplasmic motion, organelle entry, and intraorganelle movement [5, 6].

However, large cargoes delivery-with a size ranging from 10^2^ nm up to 1 μm- is still challenging since it requires large pores across the PM to avoid the contribution of endocytosis [3, 7–9]. Nowadays, the available poration strategies are based on applying external energy (electrical, optical, acoustic or mechanical) to physically open the cellular membrane [1, 3, 4, 10]. Electroporation and sonoporation have demonstrated a relatively high delivery efficiency for a wide range of cell types, allowing a complete resealing of the PM -even for large pores- with a characteristic time ranging from 1 to 10 min up to hours [11–15]. However, the use of electric and sound fields results in low cell viability and reduced reproducibility of the experiments since highly dependent on the used operational [1]. Indeed, to deliver large cargoes, electropores can be enlarged by applying longer and additional pulses, using higher field intensities, leading to a range of possible negative secondary effects on cell integrity including phenotypic changes and/or cell death [16]. Therefore, a delivery method that meets the needs of a non-invasive, scalable (e.g. large pores for large cargoes), high-throughput, easy-to-use, and cost-effective process is yet to be identified.

To date, microfluidics enabled powerful new solutions to provide highly performant poration procedures, addressing the mentioned requirements. Typical examples are the constriction-based cell squeeze constrictions, acoustic-waves based platforms, inertia-driven elongational flow and/or the viscoelastic compression [17–22]. Undoubtedly, these techniques show a great potential for satisfying the high cell throughput required for an efficient delivery inside cells, minimizing the endocytic contribution. However, weaknesses of these approaches include frequent clogging phenomena, multiple steps of poration to enlarge the pores, narrow ranges of appliable flow rates to have high throughput coupled with good cell viability, and a lack in control of pore localization on the PM. The latter problem has been partially solved by using mechanoporation microfluidic platforms based on a direct PM disruption localized at specific PM multiple points, to control where and how large the pores are [23, 24]. However, the used capture chambers and/or the needle design suffer for a strict dependency on cell dimension, processing a low number of cells. On the other hand, a lack control in applied force may result in abrupt and not uniform membrane deformation that could also compromise cell viability. Indeed, a pronounced PM bending results in a lipid configuration change which increases the membrane tension leading to the pore formation [25]. Moreover, such a tension increase intimately links to the cytoskeleton organization in terms of actin cortex disassembly, as well as it slows or blocks the spontaneous lipid reorganization for the PM resealing, weakening cell viability [26–28].

Thus, finding a microfluidic approach that allows to control the applied force and the resulting bending of cells is still challenging. Moreover, monitoring the bending could lead knowing where the pores are expected, therefore having information on both size and localization of them. Above all, such a kind of control would help in the ambitious effort of creating pores large enough to deliver cargoes of diameter even greater than 200 nm, at high cell viability.

During the last years, we have reported results addressing the need for a highly tunable and versatile in-flow compression of cells by means of a novel passive microfluidic technique based on biocompatible viscoelastic fluid-flows [29]. Such microfluidic conditions permit to minimize effects of high flow rates -typical of inertia-based flows- which makes it difficult a proper control of the cell position, deformation and orientation inside the microfluidic channel. Therefore, thanks to the applied pressure-driven flow and the viscoelastic properties of the encasing fluid, we were able to align, orient and uniaxially compress different cell types in a contactless manner using controlled levels of forces [29–31].

**Fig. 1:**
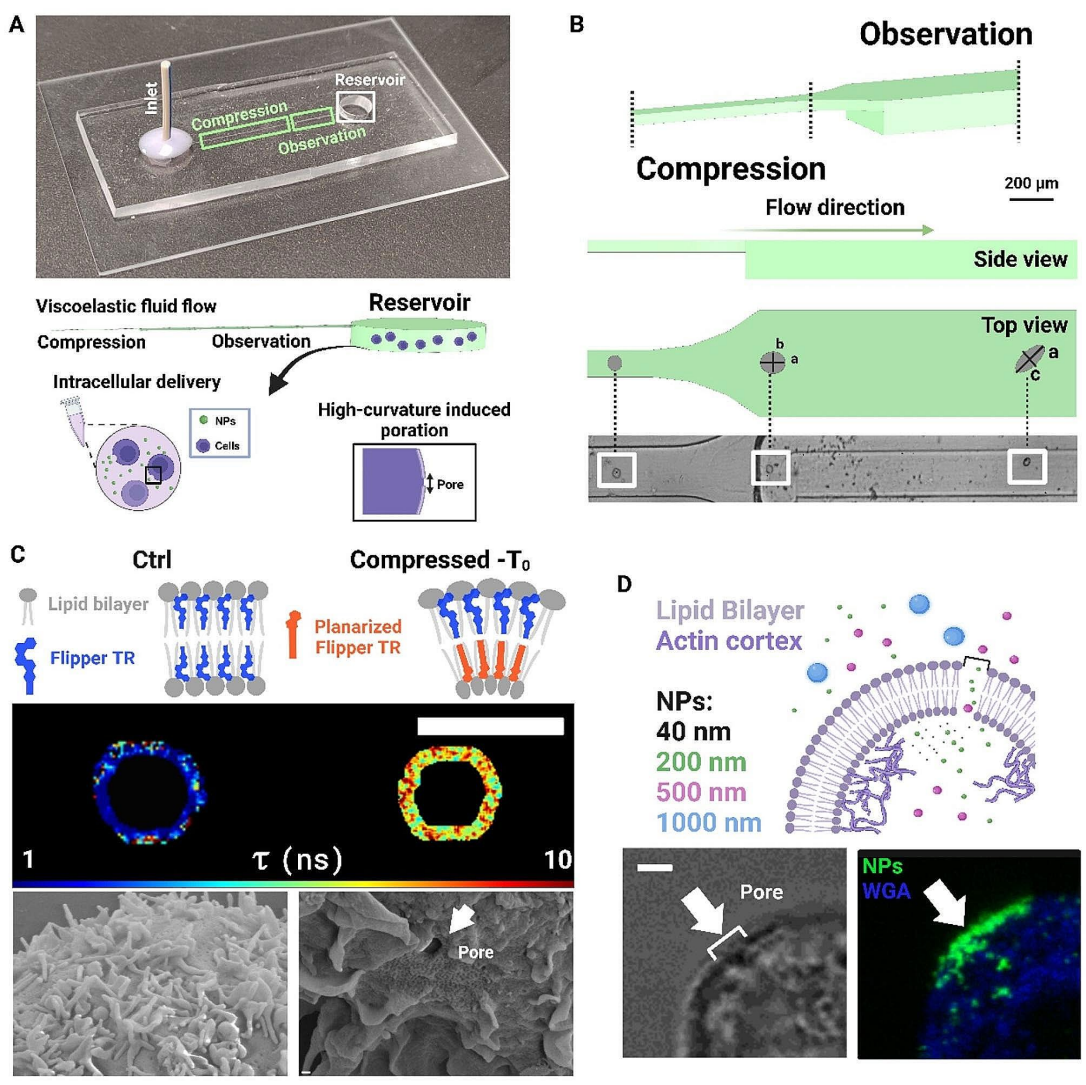
In-flow contactless compression induces high cell curvatures with resulting mechanoporation. **A** On the top, image of PDMS-glass microfluidic device with Inlet, Compression, Observation and Reservoir sections. On the bottom, a schematic of that sequence is presented. To induce the contactless compression, a viscoelastic fluid is used. From the Reservoir, cells are recovered for further analysis and passive delivery tests through the expected pores. **B** Detailing the smaller section to induce the contactless compression on cells before entering the Observation region where cells are imaged and analyzed in Top view configuration. In brightfield, a high-speed video of cells in the Observation channel were captured for the quantitative shape and curvature analysis. Scale bar 40 μm. **C** On the top, it is presented a sketch image of how the fluorophore Flipper-TR would change its configuration from a more liquid-disordered packing (Ctrl) to an increasingly liquid-ordered packing (Compressed), after the high induced curvature (Sketch created with BioRender.com). Fluorescence lifetime (τ) images of the mechanoresponsive Flipper-TR are presented, with the heterogenous mapping of tension points on the PM, from Ctrl to Compressed-T_0_, where T_0_ stands the outflow imaging step. Scale bar: 10 μm. On the bottom, by SEM acquisition, a regular ultrastructure of the PM is shown at Ctrl. Whereas, at Compressed-T_0_, a more irregular PM with a pore formation is shown. Scale bar: 0.2 μm. **D** The obtained mechanoporation drives a NPs diffusive transport trough the obtained pore. A confocal image taken at the equatorial circumference of a cell -from a 3D z-stack- with pore across the PM showing a NPs accumulation through the pore. Scale bar: 10 μm. (Sketch created with BioRender.com)

Herein, we expand upon these results by detailing new outcomes on the validation of HiViPore as a microfluidic viscoelastic approach for a one-step, localized and transient cell mechanoporation for the delivery of nano and macro cargoes (Fig. 1A). By applying the in-flow compression, we induced a high curvature at the equatorial region of cells. Such high curvature corresponds to the increase in tension of the PM, measured with a fluorescent tension reporter named Flipper-TR. Then, we spatially correlated the highest tension points with the position of the pore. Through the pores, we also tested a mechanism of NPs delivery (Fig. 1A). In details, one complete microfluidic race takes 1 min, and the throughput is ~ 100 cells/min. Cells are loaded into the device where they are aligned (Inlet) and forced to cross a small rectangular section -with a confined height- where a uniaxial viscoelastic contactless compression occurs (Fig. 1A) [29]. Then, cells move into an enlarged section where the force is relaxed, and the resulting cell deformation is measured thanks to specific in-flow rotation dynamics (Fig. 1B) [29, 31]. Finally, to test the resulting mechanoporation process, we delivered different sized NPs inside the cells recovered from the reservoir (Fig. 1D).

From a spherical-like cell shape (Ctrl-condition, Fig. S1 (i)), the applied uniaxial compression was conceived to induce a recoil along the z-axis and an equal extension in all orthogonal directions of the cell, with a final oblate spheroid shape (Compressed-condition, Fig. S1 (ii)). Along the z-axis, cells flatten, and the cell walls bend, becoming thinner [32]. In general, the bending is described by the curvatures of a plane passing through the resulting oblate-shaped PM of the deformed cell [25]. From literature, it has been demonstrated that -inside a PM- heterogenous groups of lipids tend to re-arrange and pack where the induced curvature guarantees the less energetic demanding state [33–36]. The induced bending -where the higher positive curvature is registered- and the increase in tension lead to a crack of the PM, causing the formation of a pore [25, 33, 35]. Indeed, we were able to measure high tension levels displayed in specific portion of the cell PM, where the pore locates (Fig. 1C). Nevertheless, high cell viability, evaluated in terms of both vitality and functional responsiveness, was assessed.

Therefore, the non-invasive mechanoporation allowed us to track a diffusive delivery of different NPs (Fig. 1D) through the created pores. The nanomaterial sizes are comparable to those typical of nano and macro cargoes like NPs for molecular imaging, therapeutic and/or diagnostic approaches. The chosen dimensional range goes from diameters of ~ 44 nm for smaller cargoes (like RNPs, mRNA) to larger sizes of ~ 500 nm (like carbon nanotubes and DNA plasmids) (Fig. 1D) [3, 37]. Since the obtained increase in membrane tension leads to a reduced endocytic contribution, less NPs result to be trapped inside vesicles [38, 39]. Indeed, NPs exhibiting free Brownian motion inside the cells are observed, even in the case of larger ones.

Compared to existing techniques, our approach allows not only to apply a mechanoporation on cells at high performances -in only one-step compression procedure and guaranteeing a good cell viability- but also to create large pores for delivery of large cargoes that are still challenging to passively be diffused inside cells.

## Methods

### Viscoelastic alignment and compression forces

To induce compression of single cells under viscoelastic flow conditions, we use a highly biocompatible polyethylene oxide (PEO, 4MDa, Sigma Aldrich). Fluid rheological properties have been previously investigated and properly characterized [29]. We decided to work with the so-called PEO 05 concentration, which corresponds to a polymer concentration of 0.53 wt%. We calibrated the initial pressure (~ 1000 mbar for an applied flow rate of ~ 0.05 µl/s) and velocity conditions into the microfluidic device, as well as the channel geometry to align and compress cells in a contactless way. As already published elsewhere, we released an estimation of the applied viscoelastic compressive force (Table S1) coming from the channel walls, supposing the cell already at its own equilibrium position where the competing viscoelastic and drag forces are balanced [29]. Of note, the microfluidic device is designed for tunable applied contactless compression forces in gradient direction (top and bottom), with negligible forces coming from the side walls. We computed viscoelastic compression (F_ECompr_) values, supposing the cell already at its own equilibrium position where the competing viscoelastic and drag forces are balanced [29]. The compression scales as follows,

$$\:{F}_{ECompr}\propto\:{\beta\:}^{3}\eta\:\lambda\:{U}_{Max}^2$$

where β is the confinement ratio given by cell diameter and compression channel height, η and λ are the fluid viscosity and relaxation time, respectively. U_Max_ is the maximum velocity of the fluid inside the cross-section. Tuning the rheological properties and the geometry of the channel, it is possible to change the applied compression.

### Experimental setup for the in-flow measurement

The setup includes a pressure pump (P-pump, Dolomite Microfluidics), a round shaped flexible fused silica capillary tubing (Molex) an ad-hoc designed microfluidic device, a fluid connector (N-333, IDEX) and an inverted microscope (X81, Olympus) with CMOS camera (ORCA FLASH 4.0, Hamamatsu Photonics K.K.). Briefly, a pressure pump pushes the sample volume through the capillary -where cells perfectly align- in the microfluidic device inlet, while at the end of the device a reservoir collects cells.

For the device fabrication, PDMS was employed owing to the simplicity of molding it in various shapes along with its inherent properties (transparency and biocompatibility). We used a replica molding approach which consists in the production of a 3D master where a layer of liquid PDMS is poured and cured by temperature [40]. The master was fabricated using micro-milling by carving the device structure out of a PMMA sheet. A 10:1 solution of PDMS and cross-linking agent was prepared. A layer of 3 mm of PDMS was then poured onto the mold and put in the oven at a temperature of 80 °C for 2 h for the curing process. The solidified PDMS layer was peeled off and cleaned. At this point, the first replica of the master was a negative of the desired microfluidic structure and it was replicated again to obtain the final device. The first replica was first treated with oxygen plasma (50 W–30 s) and then immersed in ethanol 100%, placed 30 min under vacuum and then 30 min at 80 °C to allow ethanol to evaporate. The treatment was performed to allow the second replica to detach from the first one once cured. At this point, a new layer of PDMS was poured onto the first replica and solidified at 80 °C for 2 h to obtain a positive replica of the microfluidic device. The obtained PDMS was then punched with biopsy punches to create the holes, for the fluidic connection (1.5 mm) and the outlet. The obtained replica was then bonded to a glass slide to obtain the final microfluidic device. The bonding was performed by means of oxygen plasma treatment, which consists in the exposure of both the PDMS and the glass slide to oxygen plasma at a power of 50 W for 60 s (CESAR ^®^ RF power generator, Advanced Energy Industries, Inc.). Then, PDMS was gently pressed onto the glass slide to complete the bonding process. At this point, a cylindrical tube was attached to the fluid inlet with a biphasic glue.

### Cell culture

We have investigated MCF-7 and MDA-MB-231 cells, kindly donated by Daidone’s group and Dr P.F. Cammarata (Institute of Molecular Bioimaging and Physiology, IBFM-CNR, Cefalù (PA), Italy), respectively. MFC-7 cells are cultured in Eagle’s minimum essential medium (EMEM, Sigma- Aldrich) containing 10% fetal bovine serum (FBS), 100 µg/ml l-glutamine and 100 U/ml penicillin/streptomycin. MDA-MB-231 cells are cultured in a 1:1 mixture of Dulbecco’s modified essential medium (DMEM, Euroclone) and Ham’s F-12 medium (Microtech) supplemented with 10% FBS, 1% non-essential amino acid mixture and 100 U ml − 1 penicillin/streptomycin.

Furthermore, cells have been checked for Mycoplasma infection using Hoechst 33,342 (Life Technologies) DNA staining. We did not observe the presence of stained dots outside the nuclei by using an inverted microscope (X81, Olympus) equipped with a water immersion objective (60 × objective with NA 1.35), showing no evidence of Mycoplasma infection.

### Measurement procedure

We defined different experimental steps. The control condition (Ctrl) corresponds to all the investigations performed to cells without in-flow compression treatment. Then, cells are forced to pass inside the microfluidic device -where PEO 0.5 flows- and recovered (Compressed). The first measurement step is performed after 1 min (T_0_) from the in-flow race and the second waiting 10 min more (T_1_). Before starting, we verified the osmolarity of our viscoelastic solution to be sure that there are no influences on plasma membrane ruptures due to alterations of osmolarity concentrations. In general, cell osmolarity ranges from 275 to 325 mOsmol [41, 42]. For this reason, the used EMEM medium was kept constant to an osmolarity range of 270–320 mOsmol. Thus, for our PEO 0.53 wt% solution, we were able to keep constant an osmolarity concentration of 274.001 mOsmol, resulting from the dilution of PEO with PBS (1×). Therefore, no influences coming from an unbalance of osmoles inside, and outside cells is expected. Moreover, adding the same PEO 0.53 wt% concentration to the medium on un-compressed cells, we already noticed that no structural alterations occur and that the obtained results are only ascribable to the in-flow viscoelastic action (Table S1) [30]. Then, we performed in-flow cell diameter measurement. We start from cells diluted in 500 µl of the viscoelastic medium to reach a final cell concentration of circa 50 cells per µl. In a typical experiment duration of 60 s, ~ 3 µl of cell suspension (concentrated to be 10^6^ cells/ml in the stock solution) are pushed through the chip and investigated by the imaging system, resulting in a total amount of ~ 100/measurement. To enable a versatile tracing of cell diameters, we used a 10x objective and a field of view of 2048 × 200 squared pixels (0.625 μm/px), which covers a final cell tracing length of 1.33 mm in the beginning of the Exit region. After cell compression, we recovered them from the reservoir at 1 and 10 min after the exit for the off-chip investigations.

Plasma membrane tension measurements were performed with a fluorescence lifetime imaging microscopy (FLIM) imaging technique. FLIM was supported by a confocal microscope (TCS SP5 II, LEICA), equipped with a 63× (NA 1.4) oil-immersion objective. Then, the image acquisition of the actin cortex was performed using a 63× oil immersion objective (NA 1.4) of a confocal laser scanning microscope (LSM 710, Zeiss). For the cytoskeleton imaging, a 20× objective (NA 0.8) of the same confocal microscope was used.

The image stacks for the NPs intracellular delivery analysis and colocalization were collected at 0.35 μm Z-spacing using a 63× oil immersion objective (NA 1.4) of a confocal laser scanning microscope (TCS STED CW, LEICA).

Instead, for the image stacks of 44 nm NPs, the intracellular delivery analysis was collected at 0.15 μm Z-spacing -because of the small NPs size- using a 63× oil immersion objective (NA 1.4).

Image resolution was fixed at 512 × 512 squared pixels with a 4× zoom factor. Particle tracing experiment was performed in time-lapse for 20 s with a 0.5 s sampling time by using a wide field fluorescence microscope (Olympus Cell-R) and a 60× water immersion objective (NA 1.35), plus 1.6× magnification of internal microscope lens.

### Viability test

The viability test was performed in a Neubauer chamber counting the number of alive cells after the microfluidic race. Dividing the number of total unstained cells by the total number of stained and unstained cells, we get 86% of viable cells after applied compression at T_0_ time. Moreover, an investigation on nucleus integrity was performed. Thanks to a DNA staining with Hoechst 33,342 and using a wide field fluorescence microscope (Olympus Cell-R) and a 60× water immersion objective (NA 1.35), plus 1.6× magnification of internal microscope lens, we were able to observe intact cell nuclei without altered morphology. Then, an acquisition of cells after 6 h from the in-flow compression was also executed, with a good recover of the adhesive functions of cells onto µ-Slide 8-well glass surfaces (Ibidi GmbH, Germany). For all the experiments, a triplicate test was performed to ensure reproducibility.

### Proliferation and functionality tests

To evaluate cell proliferation and functionality, 8-well chamber slide (Ibidi) were pre-coated with bovine type I collagen (Sigma-Aldrich, C4243) at a final concentration of 50 µg/mL in 1× PBS at 37 °C for 1 h. Then, both MCF7 and MDA-MB-231 cells in Ctrl and after Compression were seeded into separate wells of 8-well chamber slide and allowed to adhere overnight. The day after, cell nuclei were stained Hoechst 33 342 (1:5000 dilution in 1× PBS, Life Technologies) for 15 min at 37 °C. Cells were washed with 1× PBS and new fresh media was added to each well. After 24 and 48 h from cell seeding (indicated as 0-Ctrl and 24 h time points), at least 4 non-overlapping images of stained cell nuclei were taken per each condition with an inverted microscope (X81, Olympus) using 10× objective and CMOS camera (ORCA FLASH 4.0, Hamamatsu Photonics K.K.). Fluorescent images of cell nuclei were imported into ImageJ software (NIH, USA) for postprocessing and analysis. Briefly, fluorescent images were converted to binary images by using the thresholding command and nuclei’s clusters were separated by applying the watershed plugin. Finally, automatic nuclei counting was performed by using the menu command Analyze › Analyze particles. The average value of nuclei number was calculated for each sample (MCF7 and MDA-MB-231 in control conditions and after flux) and for both times (0 and 24 h). For each cell line and for each condition, the average value was normalized to the corresponding average value at the time 0. Standard deviations were calculated via error propagation.

### Osmotic shock experiments

To calibrate the tension measurement with Flipper-TR, we decided to induce osmotic shocks on a part of the cell sample. Such shocks were applied by changing the PBS concentration. We estimated an isosmotic condition with a PBS at 1× concentrated (~ 274 mOsmol) while an hypoosmotic condition with PBS at 0.5× concentrated (~ 137 mOsmol). Then, to have comparable experimental conditions with the other steps, we fixed cells both in isosmotic and after the hypoosmotic shock. As reported in literature, an increase of tension is observed in hypoosmotic condition (Fig. S4, Additional file) [27].

### Cell fixation and staining

For the plasma membrane tension measurement, a Flipper-TR (SC020- Spirochrome) functionalization was performed starting from a dilution of the stock in 50 µl of dimethyl sulfoxide, to get 1mM solution. We diluted the Flipper-TR solution to a final concentration of 1:1000 µl inside the cell suspension. Incubation of the cells at 37 °C for 40 min is completed. At the end of incubation time, cells were loaded into the microfluidic device. A part of the sample was not employed for the in-flow compression since it was used for the Ctrl condition. After compression (at T_0_ and T_1_) as well as cells at Ctrl, cells treated with Flipper-TR were fixed with 4% paraformaldehyde (PAF-Sigma- Aldrich) for 15 min at room temperature, then rinsed twice with PBS. This helped in the FLIM acquisition. Moreover, since cells are in suspension, we performed a Poly-L-lysine treatment onto µ-Slide 8-well glass surfaces (Ibidi GmbH, Germany) to allow for cell interaction with the surface and stabilization after cell sedimentation. The same staining, fixing and coating procedures were carried on for the isosmotic and hypoosmotic tests to validate the plasma membrane tension measurement (Fig. S4, Additional file). For the Wheat germ agglutinin (WGA) staining -used for cell volume measurement and colocalization analysis- alive cells in suspension were incubated at room temperature for 30 min with a WGA solution at a final concentration of 1:200 µl. Then, a part of the sample was fixed for the Ctrl condition and the other part was kept alive for the in-flow compression treatment. For immunostaining of actin cortex, cells were firstly fixed with PAF for 15 min at room temperature, then rinsed twice with PBS. Secondly, permeabilization -with 0.1% Triton X-100 (Sigma-Aldrich) for 5 min- was performed. The staining of F-Actin components -both as cortex and well-defined cytoskeleton- was performed with Alexa 488 phalloidin (Invitrogen) at 1:400 dilution for 1.5 h.

### Nanoparticles (NPs) for intracellular delivery

Green, fluorescent polystyrene nanoparticles of different diameters 44 nm, 200 nm, 500 nm and 1000 nm were purchased from Thermofisher Scientific (0.04 μm-diameter, Cat. No. F10720) and Polyscience (0.2 μm-diameter, Cat. No. 09834-10, 0.5 μm-diameter Cat. No. 17152-10 and 1 μm- diameter, Cat. No. 15,702, Polysciences). NPs are carboxylate and then negatively charged (zeta potential − 63mV). We performed two intracellular delivery experiments at the final concentration of 0.03% v/v of NPs in complete growth medium. For the 44 nm NPs, we decided to calibrate the used concentration imposing that the same surface portion of the 200 nm needs to be exposed to cells, since the difference in size is high and an equal number and/or concentration could lead to quantification errors.

The first experiment was performed at T_0_. Here, only NPs of 200 nm were used. Immediately after the microfluidic race, we put in contact the NPs solution for 2, 5 and 10 min. A Ctrl condition was carried on with non-compressed cells, at the same time of contact with NPs. At the end of each time, cells were rinsed five times with PBS to remove non internalized microparticles and fixed with 4% paraformaldehyde for 15 min. The same procedure was used for the delivery of 44 nm NPs.

The second one was performed at Ctrl and T_1_ then allowing a cell seeding onto µ-Slide glass surfaces. As the seeding starts, we simultaneously put in contact the NPs solution and allowed the contact for a total time of 20 min. In this way, we were able to perform the experiment of delivery at 4 °C for which a longer time of contact was necessary to stabilize a minimum condition of sedimentation and adhesion of cells on the plate, to facilitate the interaction with the NPs. Of course, the same procedure was used for the experiment at 37 °C. After incubation, cells were rinsed five times with PBS to remove non internalized microparticles and fixed with 4% paraformaldehyde for 15 min. For the latter, only NPs of 200 and 500 nm were used since 1000 nm were verified to be too large for a diffusive entrance inside the cell.

### MTT tests

MCF7 and MDA-MB-231 cells were seeded in triplicated in 24-well plates at a density of 30,000 cells/well and allowed to adhere overnight. 200 nm and 44 nm polystyrene NPs were diluted in cell culture medium at the same final concentrations used for uptake experiments of control and deformed cells and added to the cells for 5 min and 1 h. After the incubation, 3-(4,5-dimethylthiazol-2-yl)-2,5-diphenyltetrazolium bromide (MTT) assays was used according to the manufacturer’s instructions. Briefly, 500 µL of MTT labelling reagent (final concentration 0.5 mg/mL; Sigma-Aldrich) were added to each well for 3 h. Then, the supernatant was removed and substituted with 500 µL of isopropanol for 15 min at 37 °C, 5% CO2. The optical density of each well sample was determined at 570 nm using a microplate reader. A blank absorbance value (0.12 and 0.08 for MCF7 and MDA-MB-231, respectively), obtained from wells without cells, but treated with cell culture medium and MTT reagent, was subtracted from all the absorbance values. Then, the average absorbance value of cells treated with NPs was normalized to that of control cells incubated with media and cell viability was expressed as a percentage of the control. Standard deviations of normalized absorbance values were calculated via error propagation.

### Scanning electron microscopy (SEM) analysis

SEM analysis was performed both at Ctrl and T_0_ condition. The sample preparation for SEM acquisition considers the use of specific chemicals, reported as follows: Glutaraldehyde, Sodium Cacodylate, Osmium tetroxide which were purchased from Electron Microscopy Sciences (EMS, USA); ethanol which was purchased from Merck KGaA (Darmstadt, Germany). The following protocol procedure was followed: a primary fixative with 2.5% Glutaraldehyde in 0.1 M sodium cacodylate overnight at 4 °C. Washing: 3 × 10 min in 0.1 M sodium cacodylate Buffer at room temperature. Post fixative: 1% aqueous OsO4 in 0.1 M sodium cacodylate 1 h at 4 °C. Washing: 3 × 10 min in sodium cacodylate/0.1Msucrose Buffer. Dehydration with ethanol: Et-OH 30% for 15 min at 4 °C; Et-OH 50% for 15 min at 4 °C; Et-OH 70% for 15 min 4 °C; Et-OH 95% for 15 min at 4 °C (two times); Et- OH 100% for 15 min at 4 °C (3 times). After dehydration, samples were put in a CPD chamber for the critical point drying process (EM CPD300; LEICA). The dried samples were mounted on aluminum stubs containing carbon adhesives. Then, a sputtering with 15 nm of gold (by sputter coater HR208 Cressington) was performed. The imaging was carried out by Field Emission Scanning Electron Microscopy (FESEM) Ultraplas (Zeiss) at 5 kV with a secondary electron detector in a range of magnification between 1 kX and 25 kX.

### Numerical simulation

For the NPs diffusion simulation, we used the Transport of Diluted Species module of Comsol 5.3a with a time-dependent study. We studied the case of a free diffusion across the plasma membrane with and without pores caused by the in-flow compression. The pore sizes were chosen according to the measured dimensions from experimental results. Thus, we implemented both the case of a pore size of 1.5 μm and 500 nm. We imposed the temperature, the viscosities of the three compartments of interest (bulk of NPs, plasma membrane, cell). The process time was fixed at 5 min for the case of larger pore while at 20 min in the case of the smaller pore.

For the qualitative plot of the applied bending on compressed cells, we used the LS-DYNA compiler of Ansys. In detail, starting from a spherical object -made up by a core-shell structure- we hypothesized to apply the compression with two parallel bars, assigning the obtained experimental displacement in z direction. From this, the resulting descriptive plot of bending distribution was obtained.

### Curvature computation

We started to define the angular variations on the meridian and parallel sections of an oblate ellipsoid. In detail, we referred to φ = [0°;90°] and θ = [0°;360°] for meridian and parallel, respectively. Then, to compute the radii of curvature of an ellipsoid we computed relevant quantities as follows:

$$\:e=\frac{{a}^{2}-{c}^{2}}{{a}^{2}}$$

as the first eccentricity of the ellipsoid. The parameters *a* and *c* are the semi-axis of compressed cells, measured in-flow.

Then the radii of curvature on the meridian section:

$$\:{\rho\:}_{1}=\frac{a(1-{e}^{2})}{{(1-{e}^{2}{sin}^{2}\left(\phi\:\right))}^{3/2}}\:and\:{\rho\:}_{2}=\frac{a}{{(1-{e}^{2}{sin}^{2}\left(\phi\:\right))}^{3/2}}\:$$

as the radii that identify the surface portion (ds) of the oblate ellipsoid when a generic plane cuts in a parallel direction to the z-axis, invariant on θ. Then, the two curvatures were extracted as $\:{C}_{1}=1/{\rho\:}_{1}$ and $\:{C}_{2}=1/{\rho\:}_{2}$. At φ = 0°, the curvatures are the highest.

When the generic plane cuts in a parallel direction to the xy-axes, the radius of curvature is unique and invariant with φ. The relative curvature is equal to $\:1/a$.

The mean total curvature on the meridian section is:

$$\:J={C}_{1}+{C}_{2}$$

For the initial sphere, the curvature is the inverse of the radius (*r*_*0*_) -identifying the surface element ds_0_- of the sphere itself.

### Image analysis

Fluorescence lifetimes from Flipper-TR measurement were measured from image analysis with FLIMFit software. An exponential decay fitting is performed to extract the correspondent lifetimes of interest. In particular, the fluorescence emission decay curves were fitted with double exponentials, from which two lifetimes (τ_1_ and τ_2_) and two intensities (I_1_ and I_2_) could be extracted. τ_2_ only accounts for a minority of the signal, with a low number of photons. Then, the lifetime data presented correspond to τ_1_.

The identification of the high-tension points from the acquired RGB images was performed in Matlab R2023a. A self-written routine was used to isolate the red points in the range of 120 and 180 of intensity. In detail, a mask of the cell was created from the RGB to a grayscale image, to isolate the cell contour -fitting it with a circle to define the centroid and the relative cell radius- and merging it with the original image. Then, we were able to recognize the point and its own position on the cell, both in terms of x-y -representing them on a normalized cell radius from 0 to 1- and angular coordinates.

From a statistical analysis of the histogram distributions, we defined the degree range of 0-360° as divisible into 8 different parts, each made by 45 degrees. Therefore, a baseline value of 12.5% of points inside each 45-degree range was established as the one we would obtain if a uniform distribution was present on the 8 parts. Then, from the histograms, we extracted the highest counts for each bin, and we normalized it with respect to the total number of counts to understand how the frequencies are distributed. From this, the highest counts were extracted and plotted for each analyzed cell to demonstrate that most of them contain a concentration of points in only one range of 45°.

For the actin cortex -with the ImageJ software- we extracted the thickness values as difference between the major radius and the minor radius divided by two. Major and minor radii were measured by selection of outer and inner regions of interest (ROIs) thanks to the fluorescent signal of the actin. Then, we normalized the value of intensity associated to that annular ROI by the area of the actin ring itself. The NPs number was determined from the signal intensity -measured with ImageJ- associated to cells, imaged with a 3D z- stack. We considered the entire volume of a cell excluding slices from the top and bottom of the cell, because of possible signal alterations due to the background. The excluded parts were chosen computing the 10% of the major radius of each cell, then subtracting to the entire radius such percentage and then rescaling it with respect to the slice thickness. In this way, we obtained the total number of slices on which the analysis is useful. For counting, from the selected volumes, we applied a threshold -chosen from a mean intensity of four pixels depending on the considered NPs- to detect single particles or aggregates and to measure the volume that they occupy (Voxel Counter Plugin). Except for the case of 44 nm in diameter -for which only the voxel count was presented for a better readability of the result- we computed the number of objects dividing the occupied volume of the NPs with respect to the volume of a single NP. The delivery efficiency was determined as the ratio between the number of NPs which enters at T_0_ and T_1_ with respect to the number of NPs delivered at Ctrl. The colocalization analysis was performed from the counting of NPs. We detected colocalized pixels between the WGA fluorescence channel and the one of NPs, using the Colocalization Threshold plugin of ImageJ. We considered the colocalized NPs as particles invaginated by endosomal structures. The number of colocalized pixels identified was converted into the number of NPs and then divided by the total number of internalized NPs, in order to obtain the percentage of invaginated NPs on the total.

Particle tracing analysis was performed by an algorithm fully described [43, 44]. After obtaining the trajectories of NPs, mean squared displacements (MSDs) were calculated from the trajectories of the centroids of the NPs and correlated with the diffusion coefficient. In our analyses, we considered only those regression fittings with a goodness of fir R^2^ greater than 0.75. We considered as diffusive the NPs which have an MSD curve that undergoes a power law with exponent α = 1 and α < 1, respectively, whereas as actively transported, the particles with an MSD with a scaling exponent 1 < α < 2. The total number of both tracked and transported NPs was calculated.

### Statistical analysis

For the statistical analysis, significance has been indicated by p values (ns > 0.05; *p < 0.05, **p < 0.01, ***p < 0.001) computed by the application of a Kruskal-Wallis statistical test with Microsoft Excel 2019.

## Results

### Microfluidic contactless compression concept

**Fig. 2:**
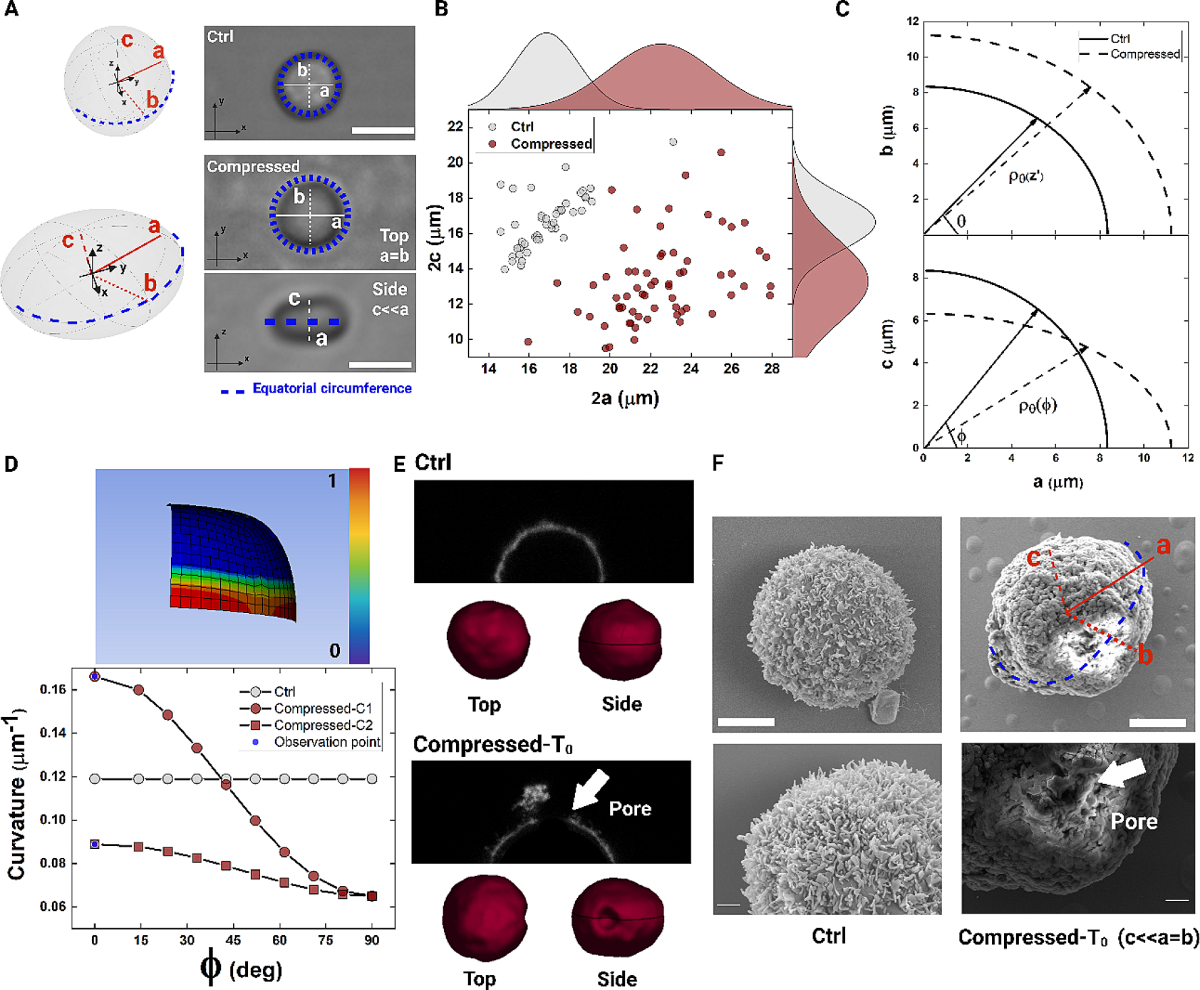
In-flow compression induces a high curvature producing large pores. **A** 3D representation and real snapshots of cells outflow and in-flow, for Ctrl and Compressed conditions respectively. From Ctrl condition, the applied compression results in cells as oblate spheroids, with the c-axis smaller than a and b semi-axes (Top view of the channel). The dashed line shows the equatorial circumference where the increasing curvature is expected. Scale bar 20 μm. **B** Scatter plots with probability distribution functions of the cell diameters are reported for Ctrl and Compressed conditions. As the 2a-axis increases, the 2c-axis decreases. **C** Polar coordinates representation of the sphere and the new oblate curves of cells, looking at the mean values of the measured cell diameters. **D** On the top, qualitative bending distribution on a cell, reported in non-dimensional quantities from 0 to 1. On the bottom, curvatures estimation for Ctrl and Compressed (C_1_ and C_2_) cells from the measure of the curvature radii. **E** Representative side view of cell with WGA staining and corresponding 3D reconstruction of cell volume using Limeseg from Fiji. For Compressed cells, at T_0_, the presence of a pore on the equatorial region is shown. **F** On the left, Ctrl cells are imaged with SEM to confirm a sphere-like shaped with regular ultrastructure of the PM. On the right, Compressed cells are presented and a pore localization at the equatorial line of the cell circumference is shown (T_0_). Top images: scale bar is 1 μm. Bottom images: scale bar is 0.2 μm

We preliminary set our approach using a breast cancer cell line (MCF-7), as a proof of concept. We fixed different experimental conditions: the control (Ctrl), the in-flow compressed state (Compressed), the T_0_-time which was immediately after the flow and the T_1_-time, waiting 10 min after flow. The level of applied compression (~ 10^1^µN for 5s) was chosen according to preserve cell functions (Table S1), as we already demonstrated elsewhere [30]. During observation, we were able to track the variation of a, b, and c defined to be the semi-axes of the cells. In this way, we monitored the changes of shape and dimension due to the applied Compression (Fig. 2A). As result, a highly pronounced change into the a and c dimension was captured, defining a resulting oblate cell shape (a = b > c, Fig. 2A and B). As the a-semiaxis increases, c decreases with a strong significance with respect to the Ctrl condition where a and c are almost equal each other (Fig. 2B). The increase of the circumferential equatorial diameter with the reduction of the third axis leads to a steeper profile of curvature at the equatorial part of cells (Fig. 2A (highlighted blue dashed line for the equatorial region) and Fig. 2B). Starting from the representation of the polar coordinates of the sphere and of the new oblate shape, we measured the cell curvature due to the compression (Fig. 2C). By cutting the oblate with planes orthogonal to the axis of rotation (z-axis), we always obtain circumferences which are characterized by equal curvature in each point, regardless of the angle of inclination (φ) and rotation (θ). Instead, if we cut with planes parallel to the z-axis, the resulting shapes are still ellipsoids with radii of curvature changing with φ (Fig. 2C and D). As result, we were able to determine a higher curvature at Compressed than at Ctrl, in the xz-plane. This was possible with the measure of the radii ρ_θ_(z’), ρ_θ1_(φ) and ρ_θ2_(φ) for the xy and xz planes. At φ = 0° (equatorial region of the cell), the curvatures C_1_ and C_2_ from ρ_θ1_(φ) and ρ_θ2_(φ) are the highest (Fig. 2D). Then, knowing the radii, we determined the total curvature as J = C_1_ + C_2_ [45]. At Ctrl, the cell is spherical, and J coincides exactly with the curvature equal to 1/a ~ 0.12 μm^− 1^. Instead, at Compressed, J is still positive and greater (~ 0.25 μm^− 1^), meaning that bending occurs in the outward direction of the cell [25]. This translates to an increase in bending energy required to deform the PM, that is forced to deviate from a ‘normal’ to a different curvature [46]. A qualitative representation of the resulting bending -as directly proportional to the curvature [47]- is presented as maximum at the equatorial region of the 3D represented oblate cell (Fig. 2D-top). The higher is the bending and positive is the curvature, more favorable the fusion of the PM bilayer is, leading to a pore formation on the equatorial region of the cell. Excitingly, 3D stack volume reconstructions of cells before and after compression (T_0_) show an evident PM interruption occurred at the equatorial part of the cell (Fig. 2E). To examine and characterize such PM poration, we decided to perform SEM acquisition. At Ctrl, PM cell ultrastructure and shape are well defined, and no defects are detectable. At T_0_, with SEM acquisition we localized pore with a diameter dimension ranging from 0.4 to 1.5 μm across the PM, at the equatorial circumference of the cell (Fig. 2F).

From SEM and 3D acquisitions, we were also able to give an estimation of poration performance, as the 70% of observed cells (total number of cells = 13) exhibit a pore on the equatorial region.

Therefore, a measure of the resulting cell viability was necessary. Always at T_0_, both a Hoechst staining to check nuclear compaction and trypan blue tests were carried out (Fig. S2A and S2B, Additional file). The latter report a mean of 86% of viable cells (n_Experiments_=3, each one of them used for mechanoporation test to ensure reproducibility of the experiment), counting still alive cells with respect to the total of collected cells (Fig. S2A, Additional file).

Some cells from the Reservoir have been plated again to observe a recovering of cell functions, showing a complete re-spreading on a glass substrate at 6 h from the mechanoporation experiment (Fig. S2C, Additional file). Moreover, a proliferation test along 24 h after the in-flow compression was carried out, denoting a not altered replication rate of cells after the applied force, compared to Ctrl conditions (Fig. S3, Additional file).

### Measurement of PM tension alterations

Dynamic changes of cell curvature or reversible poration of PM affect an inherent increase of tension related to a lipid rearrangement, heterogeneously placing themselves in both ordered and not-ordered regions [48]. Frequently, mechanosensitive probes are used to measure changes in PM tension or viscosity, reporting the distribution of different phases with different order inside [49, 50]. Therefore, to correlate the compression-induced bending and the poration of the PM with the resulting tension and lipid organization, we decided to examine the response of a planarizable push–pull probe, named Flipper-TR [34].

**Fig. 3:**
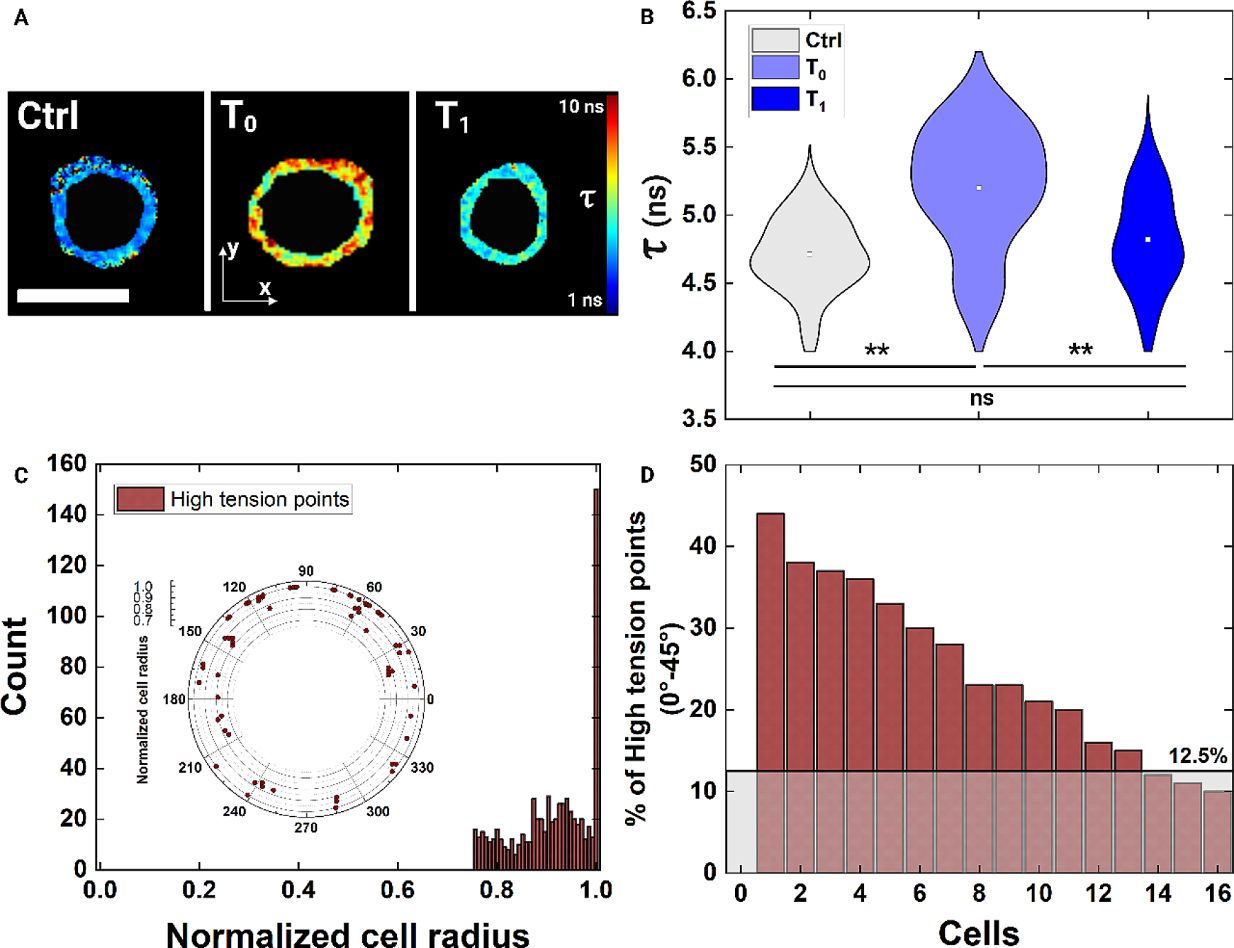
Monitoring the increase of PM tension due to high cell curvature after compression. **A** FLIM images of the PM with τ variations associated to tension increase. The colormap indicates with red the high-tension points, while in blue the low-tension points are reported. Scale bar: 20 μm. **B** Violin plots show the τ data, on the bottom. At T_0_, τ values increment while they recover the initial state at T_1_ (n_Ctrl_=18, n_T0_=16, n_T1_=10). Statistical analysis: ***p < 0.001, **p < 0.01, ^ns^*p*>0.05. **C** Histogram of the high-tension points with respect to the normalized cell radius, from 0 to 1. Most of the detected spots are at 1, the cell periphery where the PM is (n_T0_=16). The inset is a polar plot of the high-tension points of only one cell, as representative to show the points distribution on the circular cell map. **D** Percentage distribution of the high-tension points that fall into a degree range of 0–45° on cells (n_T0_=16). Up to 8 cells present a concentration of points into 45°, while the others in wider ranges from 90° up to 270°. In light gray, the baseline level of 12.5% is shown as the percentage that would be in a 0–45° range if a uniform distribution on the totality of 360° was present

To quantify relative alterations, lifetime (τ) changes were measured by FLIM acquisition on the equatorial part of cells, where the curvature is the highest (Fig. 3A). In general, high τ corresponds to an increase in PM tension. At Ctrl, τ was 4.7 ns as expected from cells in a quiescent isosmotic condition (Fig. S4, Additional file). At T_0_, τ increases with an increment of 12%, while recovers at T_1_, returning to the Ctrl values (Fig. 3A and B). Since the applied compression causes cells to flatten along c-semiaxis, we measured the localization of high-tension points (τ ~ 6.5 ns) on the equatorial section of the cell. Firstly, we showed that all the high-tension points are localized at the periphery of the cell. Indeed, referring to a normalized cell radius from 0 to 1, the relative position of most of the tension points was assessed to be around 1 (Fig. 3C and Fig. S5, Additional file). Secondly, we showed a polar distribution of the points on the periphery (Fig. 3C-inset and 3D).

Of note, we detected a preferred distribution of points through degree ranges of 45° (Fig. 3D). From the histogram plot of the high-tension points (Fig. S6, Additional file) and the identification of the bins where the highest percentage of counts falls, we found that most of the analyzed cells present high-tension points concentrated in only one degree range of 45°. The presence of a mostly unique range of degrees -on which the increase of tension can be read- confirms that the formation of one pore is expected in a certain quarter of radians on the equatorial part of cells.

### Actin cortex contribution to high curvature induced response

**Fig. 4:**
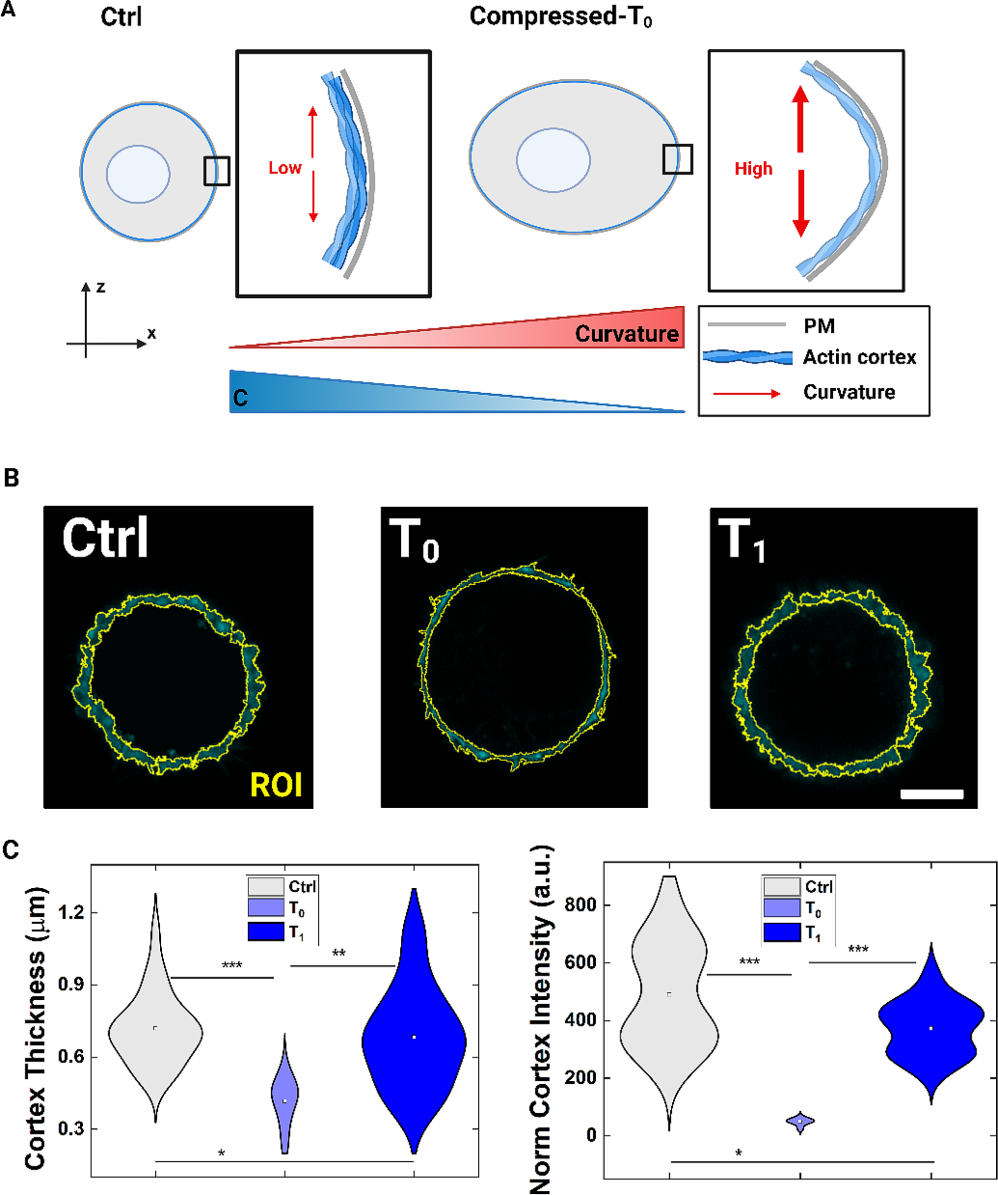
Actin cortex assembly after compression. **A** Sketch representation of how actin cortex assembly changes because of the applied compression, with the increasing curvature. (Sketch created with BioRender.com). **B** Actin cortex thickness changes at T_0_ and T_1_. ROI stands for the region of interest to which we applied the thickness measure. Scale bar: 5 μm. **C** On the left, a reduced cortex thickness is observed at T_0_. At T_1_, an almost complete restoration occurs. Statistical analysis was performed with a Kruskal-Wallis test resulting in ***p < 0.001, **p < 0.01, *p < 0.05 (n_Ctrl_=28, n_T0_=9, n_T1_=30). On the right, normalized actin cortex intensity after in-flow compression. We measured the cortex intensity normalizing that value respect to the corresponding area. Compared to the Ctrl condition, we observe a decreasing of the cortex intensity which recovers at T_1_. Statistical analysis was performed with a Kruskal-Wallis test resulting in ***p < 0.001, *p < 0.05 (n_Ctrl_=28, n_T0_=9, n_T1_=30)

Coupled with PM tension alterations, also cytoskeletal changes were investigated. In general, PM tension variation reflects on the state of the actin cortex, which is determinant for the whole cell tension and cell shape control [27, 28, 51]. Therefore, assembly and disassembly of the cortex are intimately linked with PM and the resulting actin rearrangements are responsive to changes in tension (Fig. 4A). Especially after PM poration events, such a remodeling is faster [48, 51, 52]. Along this, we measured an unfolding phenomenon of the actin when tension increases. In details, the actin cortex thickness is ~ 0.7 μm at Ctrl, perfectly matching the dimensional ranges known for MCF-7 in suspension (Fig. 4B and C) [53]. At T_0_, a cortex thickness reduction occurs of one half (~ 0.4 μm) the Ctrl value, resulting in a loss of fluorescence intensity signal (Fig. 4B and C). A recovery of actin cortex structure follows at T_1_ condition, perfectly matching a physiological recovery process duration of about 10 min for a single-cell repair (Fig. 4B and C) [54]. Thus, both cortex thickness and PM tension return to initial conditions, confirming an active repairing mechanism of cells after compression. Moreover, at the end of the proliferation test, we stained the well-formed actin cytoskeleton of cells both before and after applied compression, revealing a not altered formation of the network (Fig. S3, Additional file).

### Efficient NPs diffusive intracellular delivery

To illustrate our approach’s potential in addressing some of the current intracellular delivery challenges, we conducted several proof-of-concept experiments with synthetic NPs ranging in different dimensions and time conditions. We started from the fact that an increasing PM tension and related structural modifications of the actin cortex reduce endocytosis [38, 55, 56]. We put in contact NPs of 200 nm in diameter with cells at T_0_. Of note, NPs are rigid and negatively charged. Their dimension was chosen according to the particle/molecule dimensions currently used for therapeutic, diagnostic, and imaging purposes. To establish the best contact condition in time for the efficiency, we tracked the delivery along three different time points. Looking at the NPs_T0_/NPs_Ctrl_ ratio changes, at 2 and 5 min of contact, a burst enhancement of delivery is present. Whereas, at 10 min, the increase in NPs delivery is less since the pore and the structural compartments are repaired. This is possible since the efficiency has been computed referring each T_0_ to the respective Ctrl. Therefore, at 10 min, even the Ctrl condition presents a higher uptake and then the efficiency of delivery with respect to the initial condition decreases. However, looking at the data collected at each contact time after T_0_, a progressive increase into the NPs uptake is obtained (Fig. S9, Additional file).

**Fig. 5:**
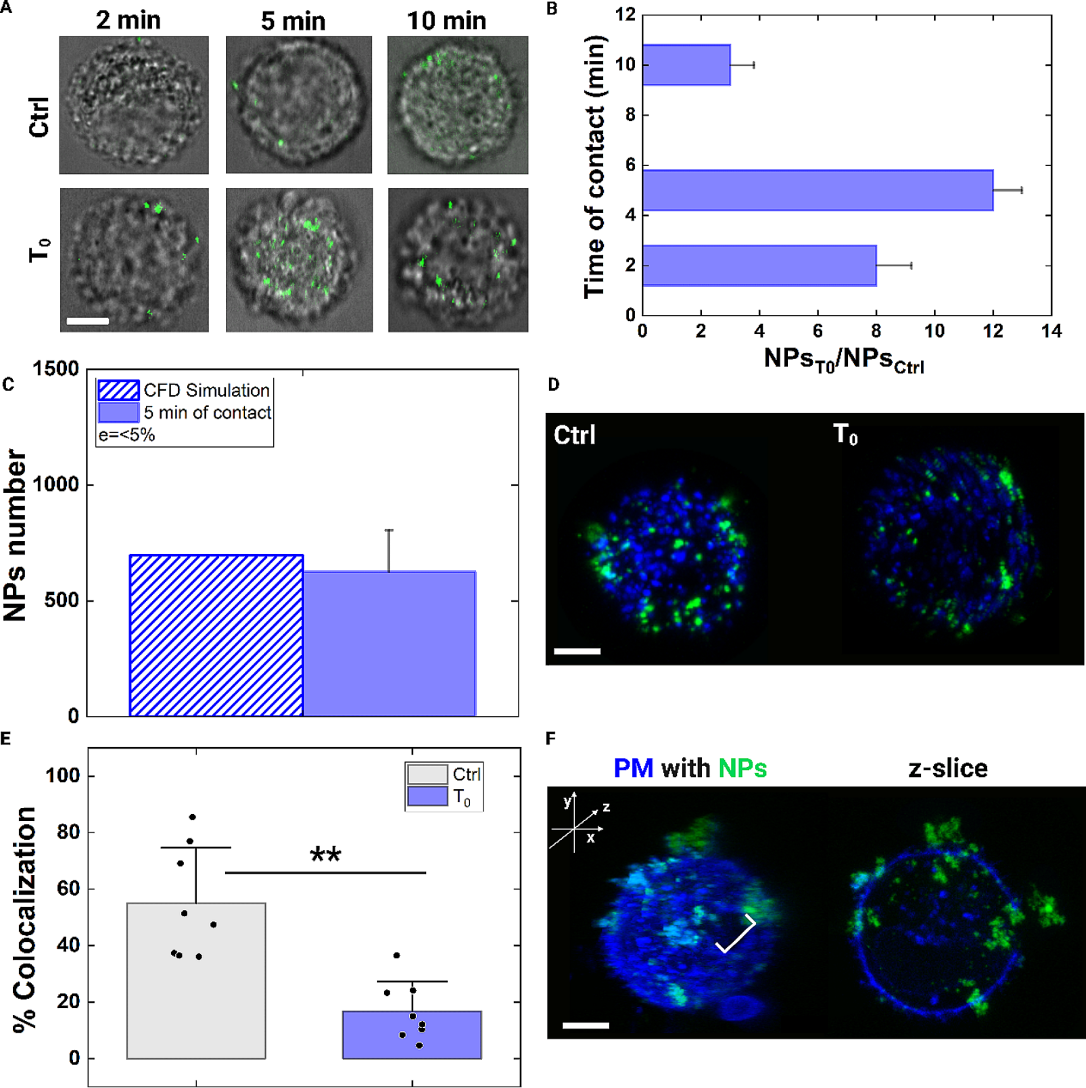
Large pores allow diffusive intracellular delivery of NPs. **A** Confocal image of MCF-7 with NPs of 200 nm at 2, 5 and 10 min of contact compared to Ctrl condition. Scale bar: 10 μm. **B** Column bar plots show the number ratio (NPs_T0_/NPs_Ctrl_) between the total number of NPs entered at T_0_ and Ctrl at 37 °C, at 2, 5 and 10 min of contact. A burst increment of that ratio is observed at 5 min. The corresponding raw data and statistical analysis are in Fig. S10, Additional file. **C** Comparison of experimental and CFD simulation results A good match among the two mean values of the results is obtained. The relative error estimated between the two results is ≤ 5%. **D** Confocal images of MCF-7 with NPs of 200 nm. In cyan, the colocalized NPs are shown. Scale bar: 10 μm. **E** Scatter box chart plot of the percentage of colocalization is reported at Ctrl and T_0_. Statistical analysis was performed with a Kruskal-Wallis test resulting in **p < 0.01 (n_Ctrl_=10, n_T0_=10). **F** Confocal 3D reconstruction of cells at T_0_. NPs accumulation is observed through the pore. Scale bar: 10 μm

As observed, the highest efficiency occurs at 5 min of contact, with an increase of 12 times in NPs intracellular entry (Fig. 5A and B, Fig. S10 Additional file). Thanks to a CFD simulation, we verified a diffusive delivery of NPs inside the cell, passing through a pore of 1.5 μm in diameter (Fig. S7 and S8 Additional file). At 5 min of contact, simulative and experimental results match with an error < 5% (Fig. 5C). Moreover, from an experimental point of view, a colocalization analysis was also performed to verify the absence of an active contribution from endocytosis. In general, endosome formation starts at ~ 10 min from the accumulation of first NPs inside the cell [55, 57]. In our case, an endosomal maturation could start during the case of 10 min of contact. At that time, we verified the amount of colocalized NPs to be ~ 14%, much less than the ~ 60% of the Ctrl condition where endocytosis occurs (Fig. 5D and E). Moreover, through the created pore, a preferential entry of delivered NPs was observed. Indeed, from a confocal 3D reconstruction, we detected NPs around the pore at the equatorial circumference of the cell (Fig. 5F).

Then, we decided to characterize a diffusive intracellular delivery also at T_1_, looking for possible differences due to the recovery state of cells. For these experiments, we decided to use three different NPs dimensions (200 nm, 500 nm, and 1000 nm). If an actual reduction in pore size is present at T_1_, then the maximum possible size of NPs capable of diffusive translocation, will give us that dimensional information. Indeed, testing the entry of different cargoes through the pore -and their subsequent trapping inside the cytosol- marks a cell as wounded or not.

**Fig. 6:**
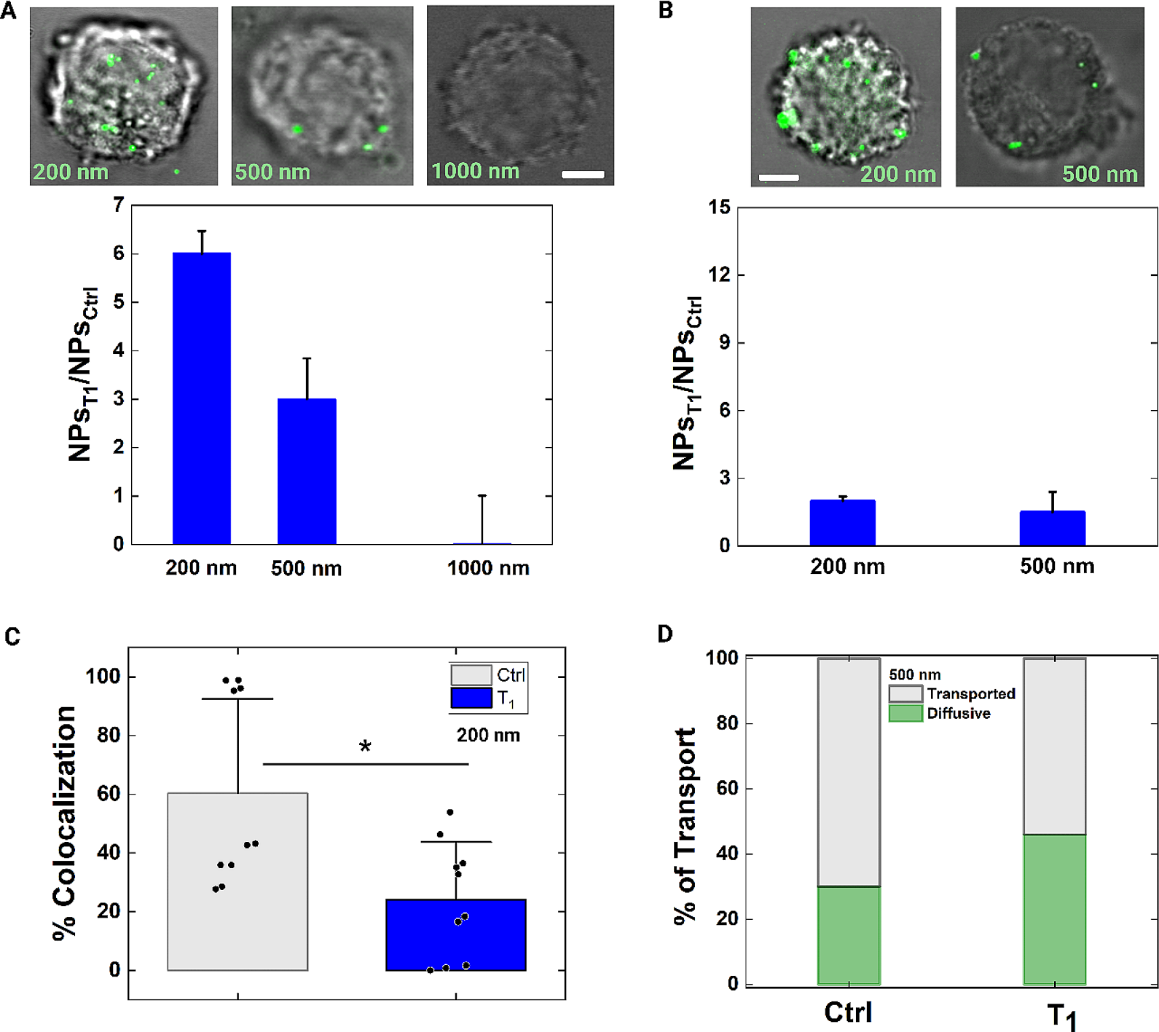
Probing pore dimension and NPs diffusion after recovery time. **A** On the top, confocal images of MCF-7 with NPs of different diameter delivered inside the cells. Scale bar: 10 μm. On the bottom, column bar plots show the number ratio (NPs_T1_/NPs_Ctrl_) between the total number of NPs entered at T_1_ and Ctrl at 4 °C. The corresponding raw data and statistical analysis are Fig. S11, Additional file. **B** On the top, representative confocal images of MCF-7 with NPs of 200 nm and 500 nm in diameter delivered inside the cells. Scale bar: 10 μm. On the bottom, column bar plots show the number ratio (NPs_T1_/NPs_Ctrl_) between the total number of NPs entered at T_1_ and Ctrl at 37 °C. The corresponding raw data and statistical analysis are Fig. S14, Additional file. **C** Scatter box chart plot of the percentage of colocalization is reported at Ctrl and T_1_. Statistical analysis was performed with a Kruskal-Wallis test resulting in *p < 0.05 (n_Ctrl_=10, n_T1_=10). **D** From particle tracing analysis, we report the percentage of NPs moving as diffusive and transported for the Ctrl and T_1_ conditions (nNPs_Ctrl_=40 and nNPs_T1_=65). At T_1_, the percentage of diffusive NPs increases

For such a kind of spatial-temporal calibration of pore size, we performed the experiment at 4 °C minimizing both the recovery mechanism and the endocytosis. For practical reasons, the time of contact was chosen to be longer than the previously used times. Our results show that the final number of NPs inside cells is still higher at T_1_ than at Ctrl. Indeed, we appreciated that the total number of delivered NPs of 200 nm (NPs_T1_) increases of ~ 6 times respect to the Ctrl (NPs_Ctrl_, Fig. 6A). For 500 nm, NPs_T1_ is ~ 3 times higher than NPs_Ctrl_. Instead, a trade-off dimension is reached for NPs of 1000 nm since they do not cross the PM (Fig. 6A, Fig. S11 Additional file). Thus, we expected that the pore dimension reduces from 1.5 μm to ~ 500 nm in diameter, on the average. To test this, a new CFD simulation was performed, with a pore diameter of 500 nm. A good match (error < 5%) of experimental and simulative results was obtained, both for NPs of 200 nm and 500 nm, confirming the diffusion of the NPs (Fig. S12 and S13, Additional file). Moreover, we performed the same experiment at 37 °C, where the delivery efficiency results less pronounced. In fact, for both 200 nm and 500 nm, the efficiency decreases (Fig. 6B, Fig. S14 Additional file). From colocalization analysis, a reduced percentage of colocalized NPs of 200 nm was still confirmed (Fig. 6C). In the case of NPs of 500 nm, we performed the measurement of MSDs from particle tracing [44]. The results show that ~ 50% of the delivered NPs move by simple diffusion inside the cell (Fig. 6D). Thus, the endocytic contribution is still reduced, although the recovery.

### NPs uptake as a function of different cell lines and NPs sizes

To demonstrate the versatility of the mechanoporation approach, we decided to manipulate another cell line, namely the MDA-MB-231 as the highly metastatic counterpart of the MCF-7. In our recent works, we already measured a high cell deformability for MDA-MB-231 [30, 58]. These cells show an actin cortex reorganization after the applied compression -as the same reported for MCF-7- eliciting an active mechanical response of the complex PM-cortex.

**Fig. 7:**
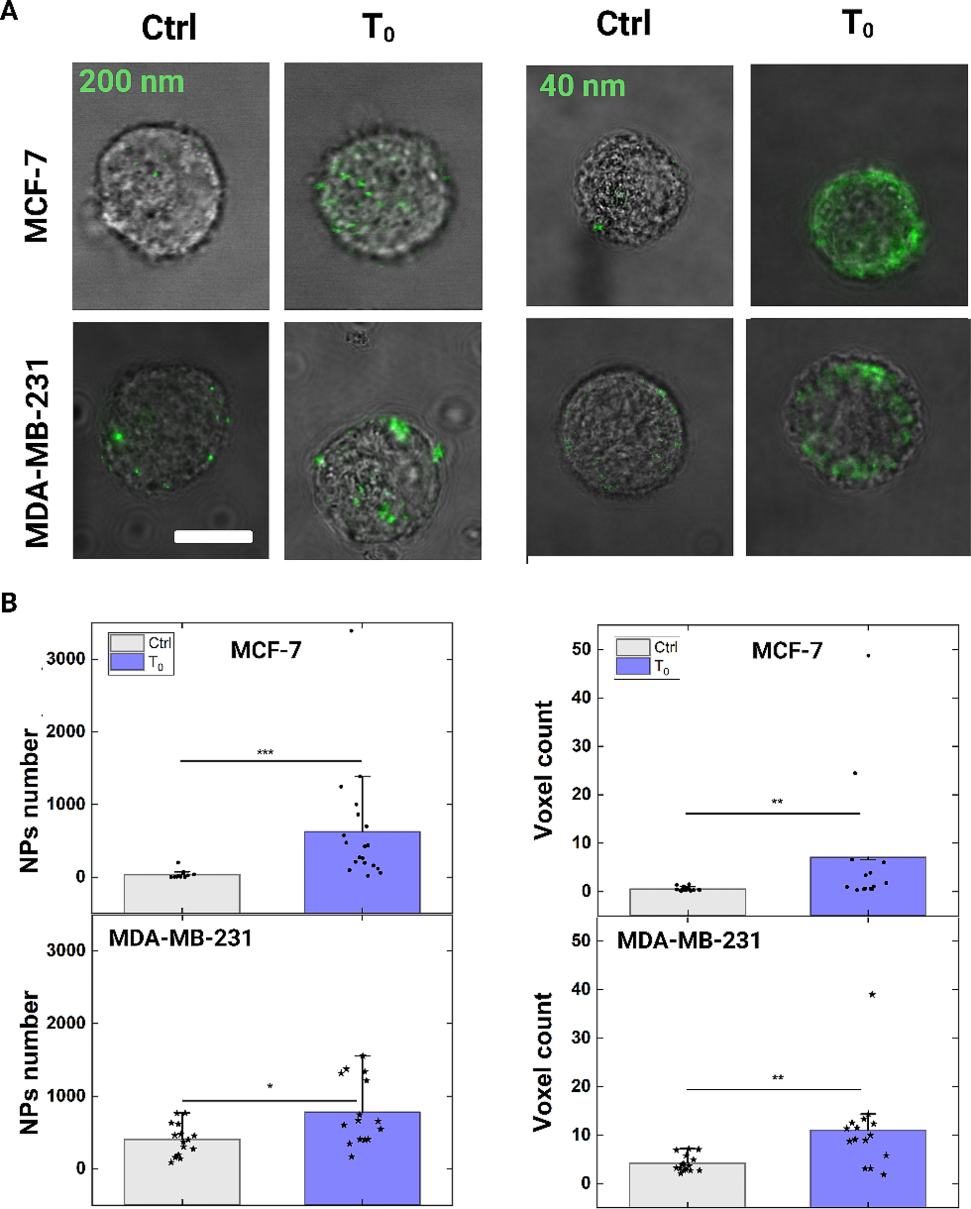
Studying the uptake of 200 nm and 44 nm NPs in MCF-7 and MDA-MB-231. **A** Representative confocal image of MCF-7 and MDA-MB-231 with NPs of 44 and 200 nm in diameter delivered inside the cells. Scale bar: 10 μm. **B** On the left, **s**catter box chart plot of number of NPs (200 nm) delivery at Ctrl and T_0_. On the right, **s**catter box chart plot of voxel count representing the amount of NPs (44 nm) delivered at Ctrl and T_0_. Statistical analysis was performed with a Kruskal-Wallis test resulting in *p < 0.05, **p < 0.01, ***p < 0.001 (n_Ctrl_=15, n_T0_=15, for both MCF-7 and MDA-MB-231 at Ctrl and T_0_)

Both MCF-7 and MDA-MB-231 passed through the device and have been recovered at T_0_. Firstly, we compared the passive NPs (200 nm) delivery inside the cells at 5 min of contact, from the best uptake condition already measured with the MCF-7. As observed, the efficiency of delivery increased by ~ 2 times in MDA-MB-231. These cells -as highly invasive- are recognized to be more prone to uptake processes [59, 60]. Indeed, already at Ctrl, NPs enter the MDA-MB-231. Therefore, the delivery enhancement is less pronounced than MCF-7 (Fig. 7).

Secondly, we tested also for the delivery of smaller NPs (44 nm in diameter), to replicate typical sizes of other cargoes like ribonucleoprotein complexes (RNPs). Indeed, RNPs range from 10 to 150 nm in diameter [61]. Also in this case, the uptake results to be increased at T_0_, with 5 min of contact. Of note, in the case of MCF-7, the delivery is ~ 12 times higher than Ctrl, as observed for the 200 nm NPs. For simplicity, we reported the quantity of 44 nm NPs as a voxel count, because of smaller NPs size. Still of about 3 times is higher the enhancement in delivery for MDA-MB-231 (Fig. 7).

Moreover, cellular functionality and absence of cytotoxic effects were tested for both 44 and 200 nm NPs. A MTT test was performed with cells in contact with the NPs both at 5 min and 1 h of time, to confirm that no side effects arise from the delivery inside the cells. A complete absence of cytotoxicity was confirmed for both the NPs sizes and cell lines (Fig. S15 and S16, Additional file). As we already did for MCF-7, a proliferation test and an actin cytoskeleton structural check also on the MDA-MB-231 was performed (Fig. S3, Additional file), without relevant changes after mechanoporation.

## Discussion

Intracellular delivery is an important yet challenging step in biological imaging for diagnostics, basic research, RNA and cell-based therapies. Commonly used cargoes for intracellular delivery span over a wide range of dimension, from 10 nm to 1 μm, thus asking even for large pores to pass through [3]. Nowadays, there are diverse options, platforms, techniques, and protocols designed for intracellular delivery. However, most of them suffer from at least one of the following issues: low delivery efficiency, low scalability, high cost, complexity in preparation, operational difficulty, loss of cell function [2].

In this work, we presented HiViPore as a microfluidic viscoelastic contactless and highly viable cell mechanoporation approach based on a controlled increase of cell curvature to induce high PM bending and tension. The technique responds to the requirements of creation of reversible large pores in controlled and biocompatible fluid-flow conditions. In details, to perform the cell mechanoporation effectively and gently, the approach adopts a one-step PM disruption strategy based on an in-flow viscoelastic contactless compression. Compared to other existing techniques still based on viscoelastic fluids -which undoubtedly address the throughput issue [19, 62]- HiViPore induces the mechanoporation without using complex geometries as well as without changes depending on the cell line. Moreover, it preserves more cell viability since it does not force cells to pass through spiral and/or extensional regions.

Thanks to a control of the resulting deformed shape, the identification of a high equatorial curvature allowed us to predict where the poration occurred. Then, SEM images indicated a final mean diameter of 0.78 ± 0.5 μm. Moreover, a spatial-temporal control of the related PM tension and cytoskeletal alterations revealed an active repairing mechanism of cells that result to be highly viable after poration. Such a process was confirmed by the complete recovery of Flipper-TR lifetime on the PM, the stabilization of the actin cortex structure and the progressive closure of the pore. In ~ 10 min, the mentioned resealing process occurred, perfectly matching the recovery mechanism times known for single cells [54]. Up to date, no one-step in-flow mechanoporation strategy enables a poration with a so large final pore dimension, guaranteeing a high cell viability of ~ 90%. Indeed, even cell-squeeze based techniques need to induce multi-step compression through constrictions to enlarge the resulting pore dimensions [24]. Nevertheless, strategies like electro and sonoporation can create pores with dimensions ranging from 20 to 500 nm but they lack cell viability [63–65].

Moreover, the successful mechanoporation enabled diffusive NPs intracellular delivery tests, independently from intracellular vesicle trapping. The approach is versatile and allows to improve passive delivery among different cell lines, using different NPs sizes. Of note, the highest possible entrance of NPs was defined to be at 5 min, when the material entrance exhibits an increase of 12 times with respect to the control condition. The evidence of an absent endocytosis responds to the requirement to have optically accessible cargoes inside the cells, avoiding any possible biological and functional alteration of the nanomaterial [5].

Moreover, the short time window presented in the study optimizes the net delivery of NPs inside the cell, by also minimizing side effects due to long times of manipulation of cells and deliverable cargoes.

## Conclusions

In summary, microfluidics offers many advantages over current standard techniques for cell mechanoporation, but they are partially limited in a tradeoff between high throughputs and high cell viability, necessity of multiple steps for obtaining large pores, easy and low-cost operational procedures. To provide a possible solution to these problems, we present HiViPore as robust and with a great potential for high-efficiency delivery of exogenous material applications useful for molecular imaging, cell engineering and therapy purposes. We demonstrated the suitability of the microfluidic technique to induce a precise and localized pore formation across the cell PM, through which a passive delivery of nano and macro particles was observed. The device has the advantages that only one step of in-flow cell compression results in a successful mechanoporation at high cell viability, and it can be easily adapted to the specific challenges of experimental needs like high throughput and online delivery of cargoes. For example, these can be realized by parallelizing the design with one pressure-driven inlet which splits into n-different outputs from which cells can be collected with resulting higher throughput. Another possibility is the use of a lower viscoelastic fluid concentration (e.g. PEO 0.4 wt%) coupled with higher applied pressures (in a range of 1000–1300 mbar) which could help in increase the throughput up to 300 cells/min for a single channel configuration. Moreover, considering commercial syringe systems with several inlets each with thousands of parallel channel sections, we could further increase the cell throughput. Finally, lateral channels -in the proximity of the reservoir- could be added to deliver the exogenous material of interest without affecting the performance of mechanoporation.

### Electronic supplementary material

Below is the link to the electronic supplementary material.

Supplementary Material 1

## Data Availability

The datasets used and/or analyzed during the current study are available from the corresponding author on reasonable request.
