# Supplementary material for: HiViPore: a highly viable in-flow compression for a one-step cell mechanoporation in microfluidics to induce a free delivery of nano- macro-cargoes
Source: J Nanobiotechnology. 2024 Jul 27;22:441. doi: 10.1186/s12951-024-02730-y (PMC11282774; doi:10.1186/s12951-024-02730-y)
Supplement: Supplementary file 1 — Supplementary Material 1 [file 12951_2024_2730_MOESM1_ESM.pdf]

Supplementary Information

Title

HiViPore: a highly viable in-flow compression for a one-step cell mechanoporation in microfluidics to induce a free delivery of nano- macro- cargoes.

Author(s), and Corresponding Author(s)*

Maria Isabella Maremonti, Valeria Panzetta, Paolo Antonio Netti and Filippo Causa*

Table S1. In-flow viscoelastic compression forces.

Cell line	Mean value of cell major diameter (µm) at initial condition (± standard deviation)	Applied in-flow force (µN)	Damage threshold in-flow force (µN) [1]
MCF-7	18.40 ± 1.010 (n_{Cell}=30)	44.00	459.00
MDA-MB-231	14.79 ± 0.240 (n_{Cell}=30)	23.63	160.00

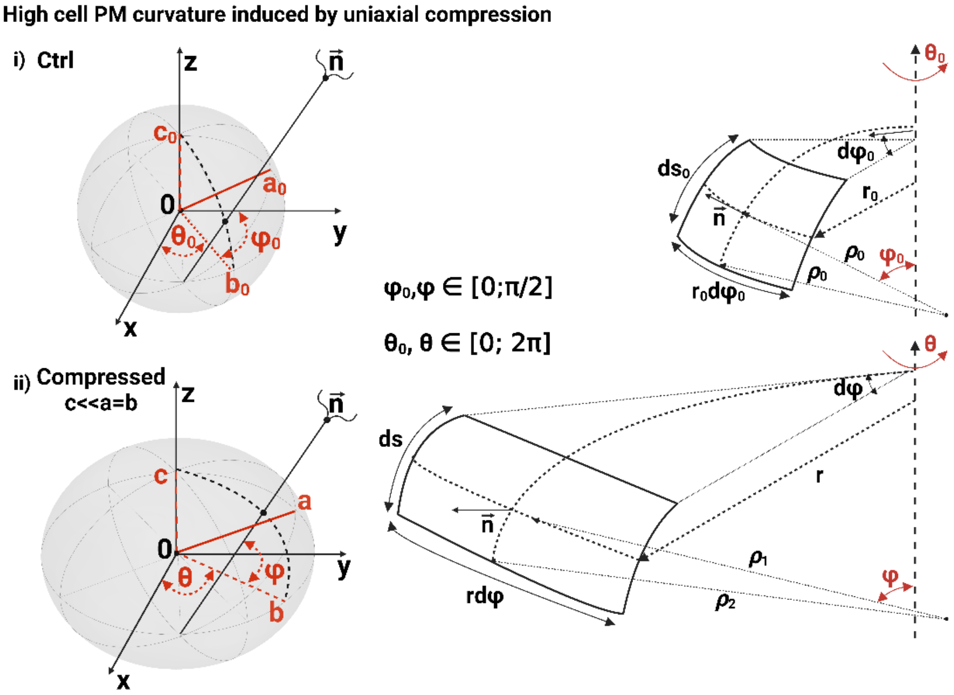

Fig. S1 Schematic of the induced high cell PM curvature for the resulting mechanoporation. A On the left, illustration of the initial (Ctrl) and final (Compressed) of a cell and a relative specific element of the PM. The three-dimensional (3D) representation of the cell shows the qualitatively a0, b0 and c0 semi-axes variation after compression where they become a, b and c. In the middle, the spatial coordinates are (θ_0, φ_0 and r_0) and (θ, φ and r) for Ctrl and Compressed conditions, respectively. The principal radii of curvature for the surface element are ρ_0 and ρ_1-ρ_2 to describe the local surface ds_0 and ds at Ctrl and Compressed, respectively. Therefore, the two principal curvatures were extracted from ρ_0 and ρ_1-ρ_2.

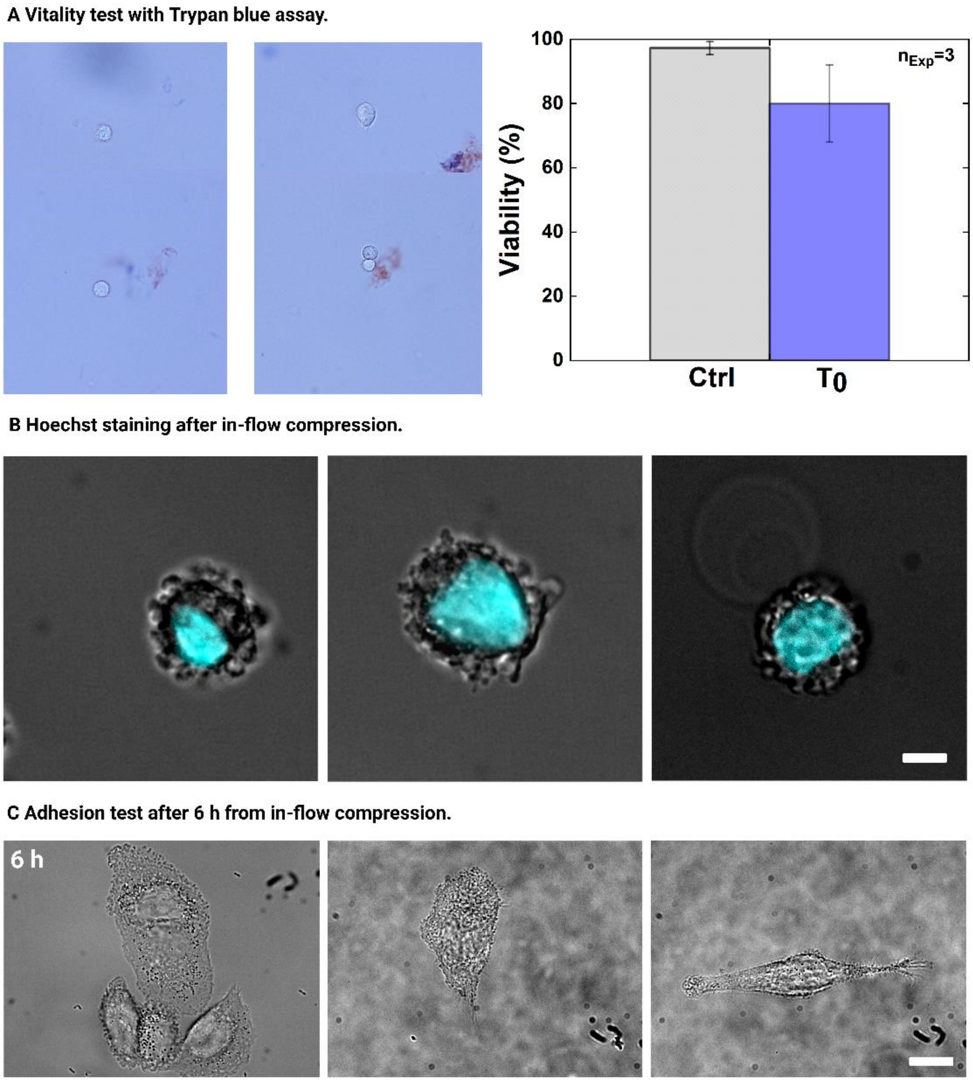

Fig. S2 A On the left, images of cells treated with Trypan Blue are shown. On the right, the assessment of the mean cell viability is reported at 86% at T_0 after in-flow compression, with a triplicate experiment ($n_{CellsCtrl}$=900, 950, 650 *per* experiment in 100 µl of final solution- $n_{CellsT0}$=100, 75, 125 per experiment in 10 µl of final solution). B To check the nucleus structural integrity, we performed a Hoechst staining on alive cells at T_0, after in-flow compression. C Adhesion test on compressed cells after 6h from the in-flow test. A complete reattachment of cells is observed on glass substrates.

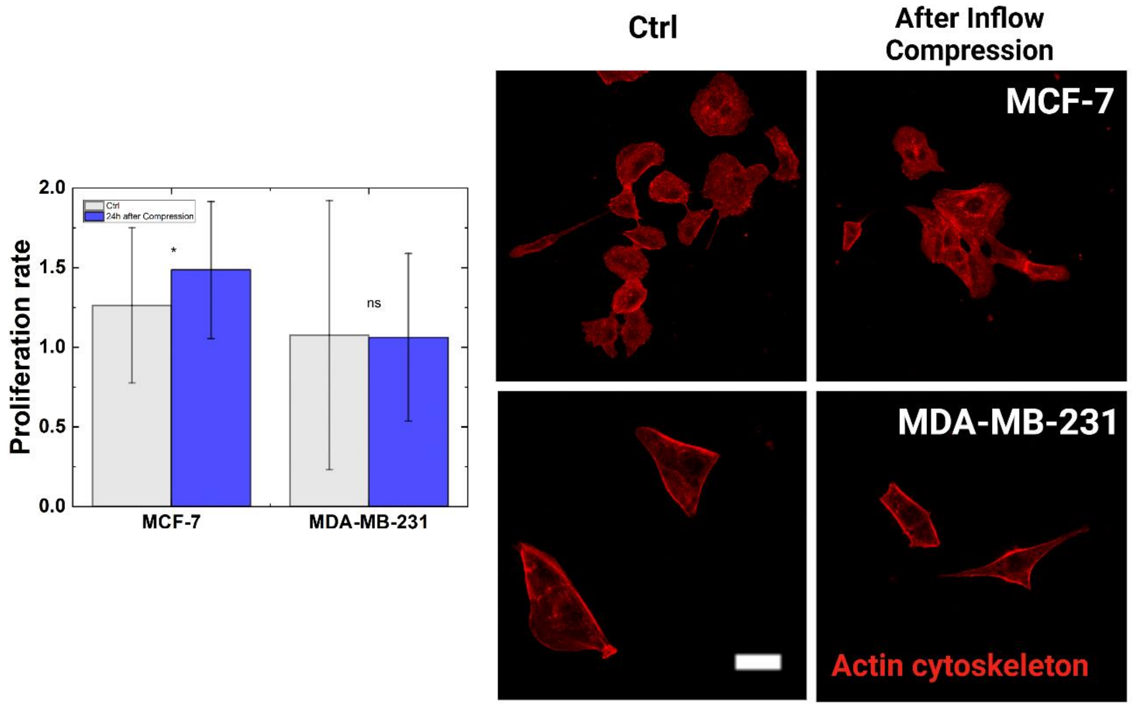

Fig. S3 Proliferation and functionality tests after applied in-flow compression. Functionality has been tested by staining the actin cytoskeleton of the used cell lines. Statistical analysis by Kruskal-Wallis test with $^{ns}p>0.05$ and $*p<0.05$. Scale bar: 10 µm.

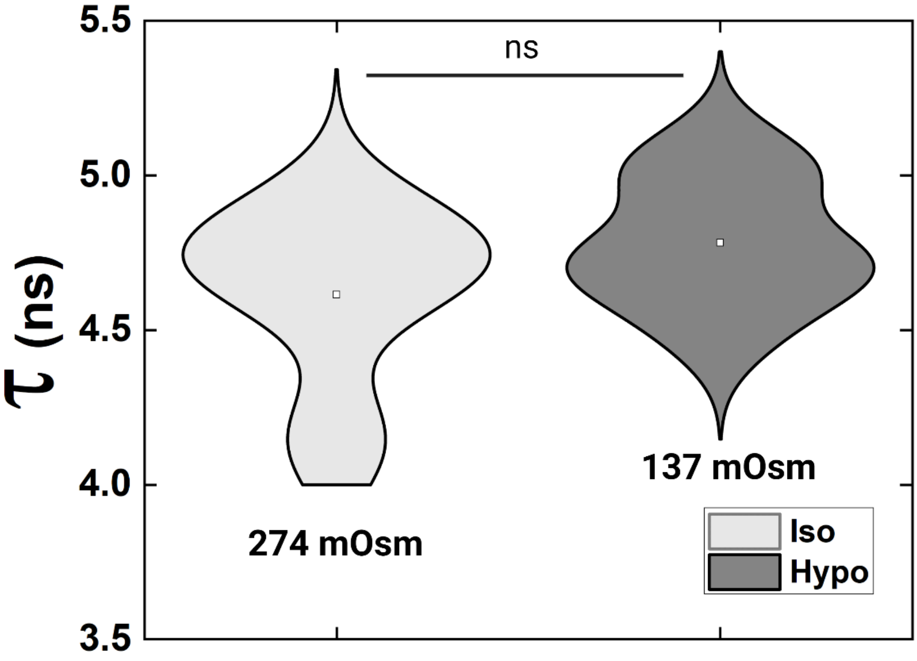

Fig. S4 Fluorescence lifetime at different osmotic conditions. The τ variation at isosmotic (Iso) and hypoosmotic (Hypo) condition is reported. We report an increase of the tension from the Iso to the Hypo case. Statistical analysis by Kruskal-Wallis test with $^{ns}p>0.05$ ($n_{Iso}=9$, $n_{Hypo}=9$).

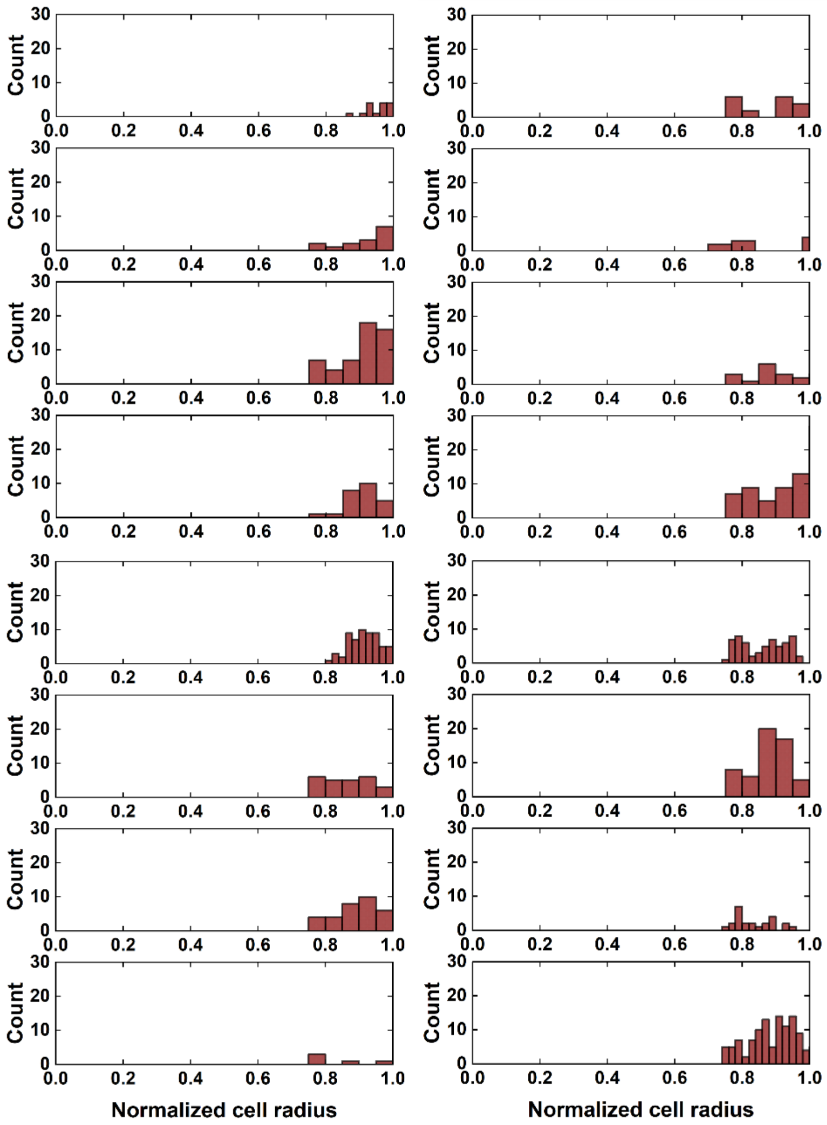

Fig. S5 Histograms of the high-tension points positions on the normalized cell radius of cells. Points in the range of 0.9-1 mean a peripheral accumulation of the points, as expected from the high membrane tension response. n_{Cells}=16 at T_0 condition.

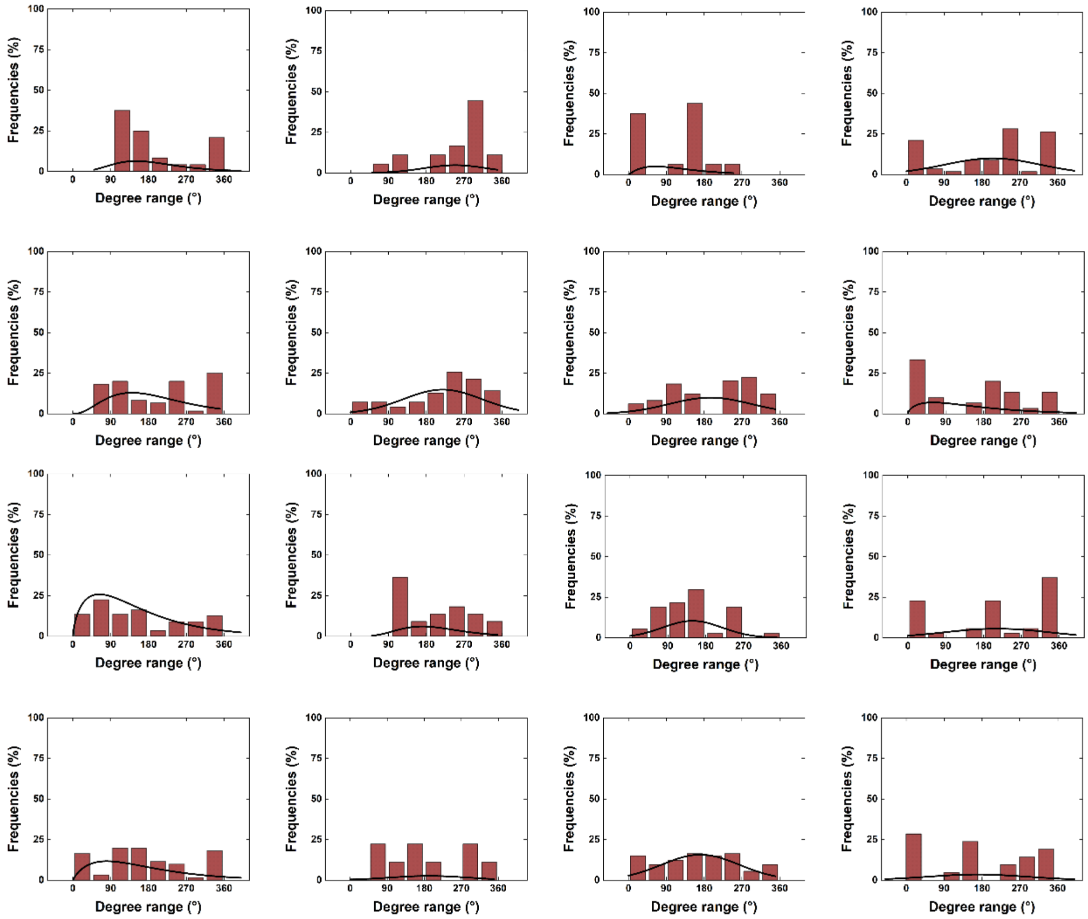

Fig. S6 Histograms and distributions of normalized frequencies of high-tension points with respect to the possible angular position on 360° of excursion. The accumulation of such points into the different degree ranges was determined defining an accumulation on preferred regions of the cells where the pores are expected. n_{Cells}=16 at T_0 condition.

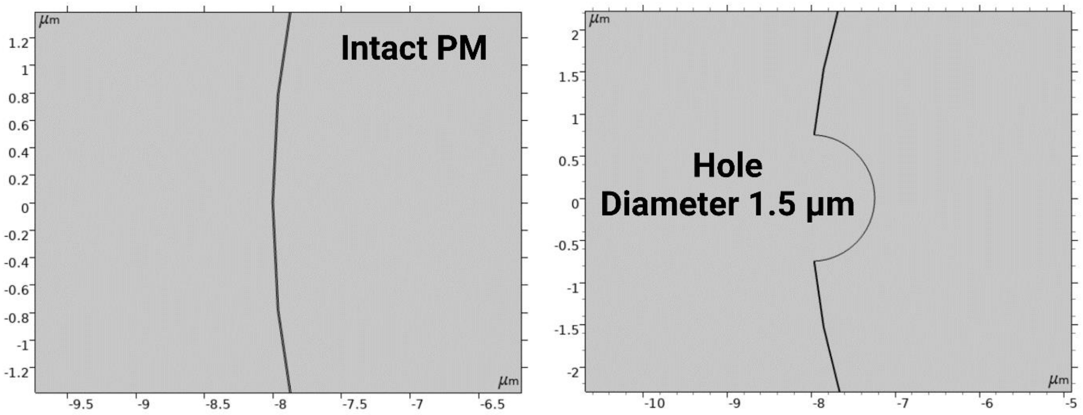

Fig. S7 Geometry domain of the COMSOL simulation. On the left, zoom-in of the plasma membrane without pores. On the right, zoom-in of the plasma membrane with pore of 1.5 μm inside.

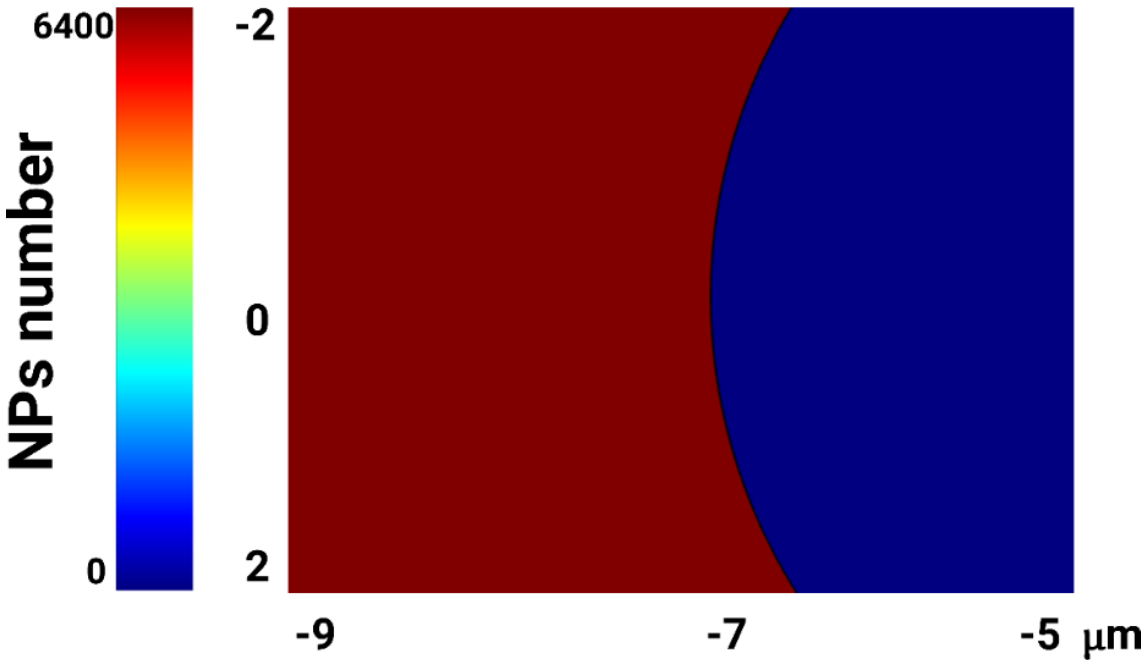

Fig. S8 COMSOL simulation of cell plasma membrane with crossing pore points. Zoom-in of the NPs number profile variation across the plasma membrane without pores.

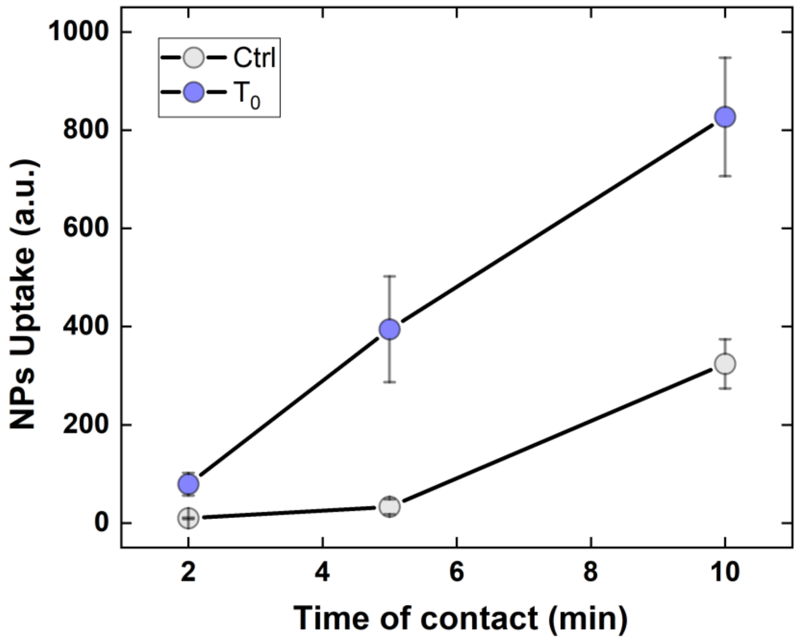

Fig. S9 NPs uptake along three times of contact (2,5 and 10 min) at Ctrl and T_0 for MCF-7. The experimental data are presented as mean and standard error (2 min: n_{Ctrl}=15, n_{T0}=16, 5 min: n_{Ctrl}=19, n_{T0}=13, 10 min: n_{Ctrl}=35, n_{T0}=30).

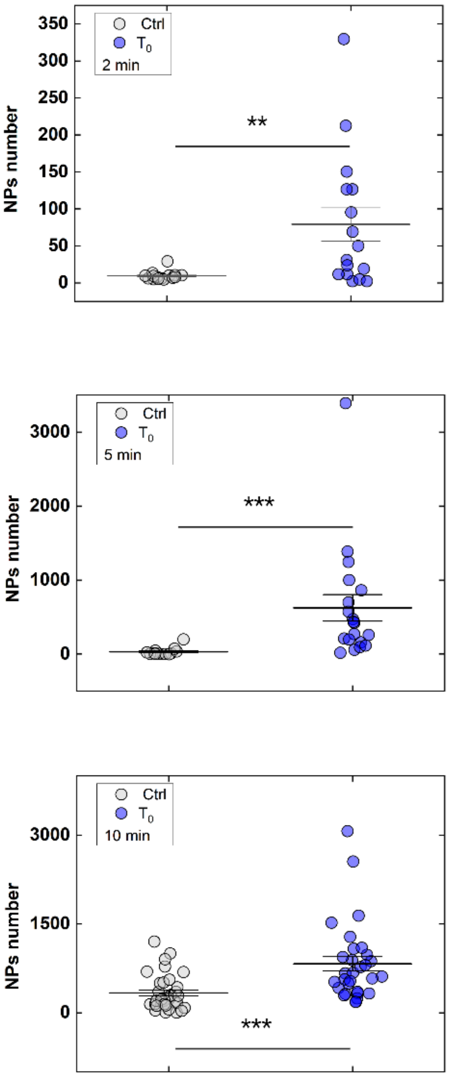

Fig. S10 NPs number after intracellular delivery at 37°C at 2, 5 and 10 min of contact. The comparison between Ctrl and T_0 is shown for NPs of 200nm. Statistical analysis with a Kruskal-Wallis test results in ***p<0.001, **p<0.01 (2 min: n_{Ctrl}=15, n_{T0}=16, 5 min: n_{Ctrl}=19, n_{T0}=13, 10 min: n_{Ctrl}=35, n_{T0}=30).

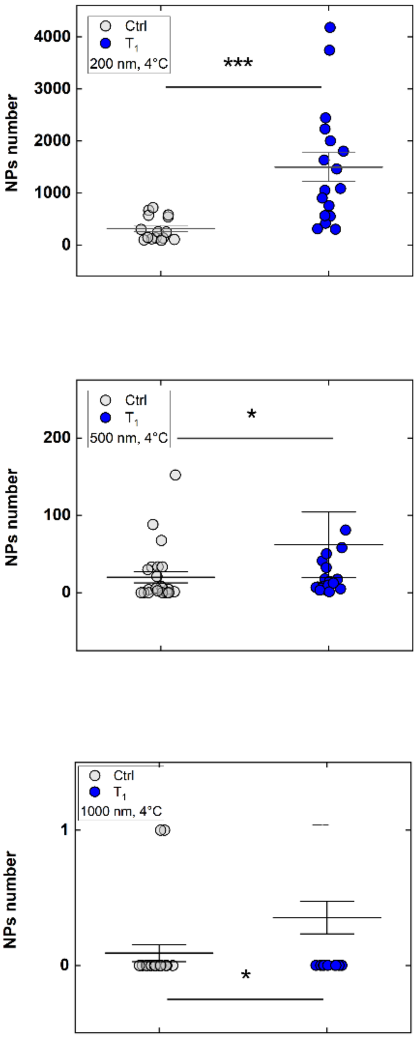

Fig. S11 NPs number after intracellular delivery at 4°C. The comparison between Ctrl and T_1 is shown for NPs of 200nm, 500nm and 1000nm. Statistical analysis with a Kruskal-Wallis test results in ***$p<0.001$, *$p<0.05$ (200nm: $n_{Ctrl}=15$, $n_{T1}=17$, 500nm: $n_{Ctrl}=25$, $n_{T1}=20$, 1000nm: $n_{Ctrl}=22$, $n_{T1}=17$).

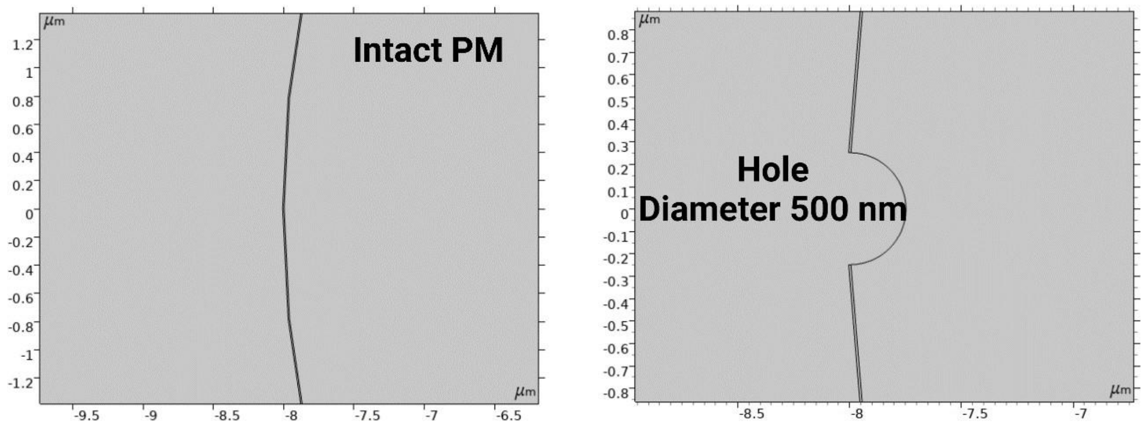

Fig. S12 Geometry domain of the COMSOL simulation. On the left, zoom-in of the plasma membrane without pores. On the right, zoom-in of the plasma membrane with pore of 500 nm inside.

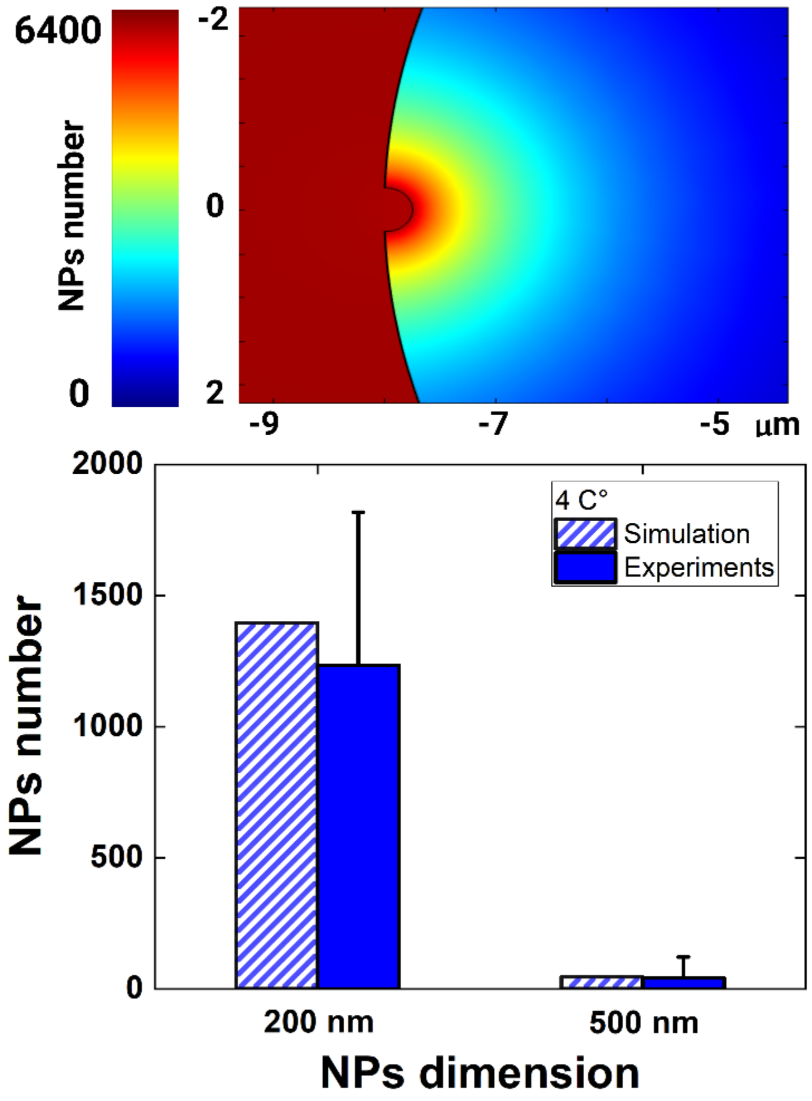

Fig. S13 On the top, zoom-in of the CFD simulation image output where we implemented a NPs diffusion transport across the PM with a crossing pore point and inside the cell. The colormap stands for the number of delivered NPs inside the cell after 20 min of contact. On the bottom, we present the comparison between the simulation and the experimental results of the intracellular delivery of NPs of 200 and 500 nm. The relative error estimated between the experimental and the simulative results is $\leq 5\%$. The experimental data are presented as mean ± standard deviation.

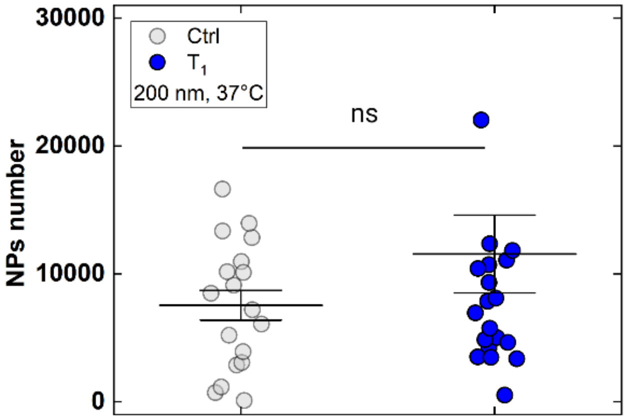

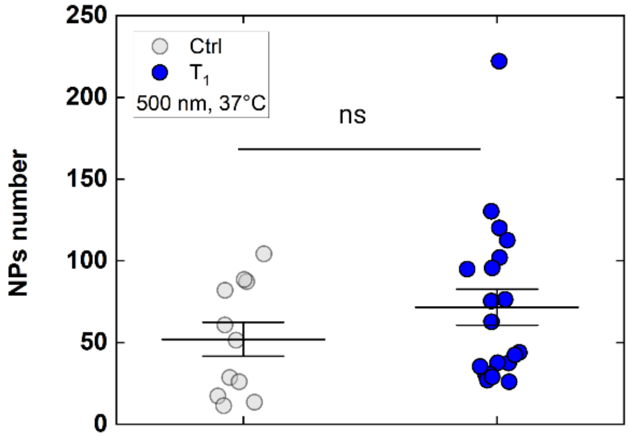

Fig. S14 NPs number after intracellular delivery at 37°C. The comparison between Ctrl and T1 isshown for NPs of 200nm and 500nm. Statistical analysis with a Kruskal-Wallis test results in $^{ns}p>0.05$(200 nm: $n_{Ctrl}=19$, $n_{T1}=21$, 500 nm: $n_{Ctrl}=11$, $n_{T1}=20$).

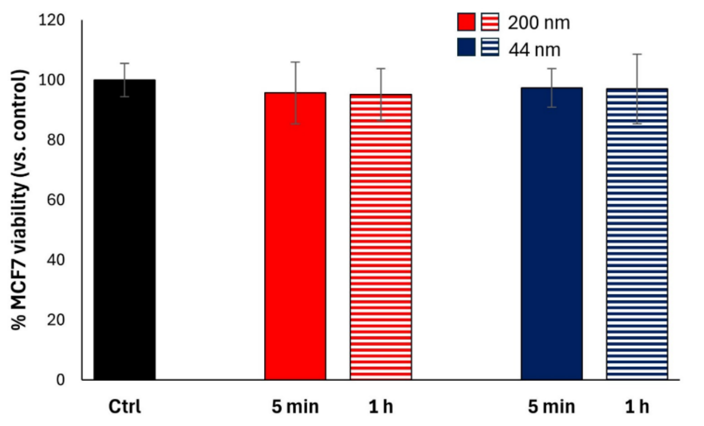

Fig. S15 MTT test for MCF-7 in contact with 44 and 200 nm NPs for 5 min and 1h. There are no statistically significant differences among the tested conditions.

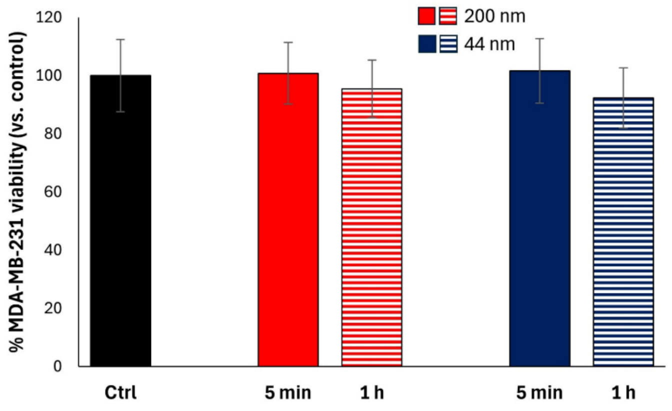

Fig. S16 MTT test for MDA-MB-231 in contact with 44 and 200 nm NPs for 5 min and 1h. There are no statistically significant differences among the tested conditions.

References

[1] M. I. Maremonti, V. Panzetta, D. Dannhauser, P. A. Netti, F. Causa, Wide-range viscoelastic compression forces in microfluidics to probe cell-dependent nuclear structural and mechanobiological responses, J R Soc Interface 2022, 19, 20210880.
